# Supplementary material for: BET Inhibition Induces HEXIM1- and RAD51-Dependent Conflicts between Transcription and Replication
Source: Cell Rep. 2018 Nov 20;25(8):2061–2069.e4. doi: 10.1016/j.celrep.2018.10.079 (PMC6280123; doi:10.1016/j.celrep.2018.10.079)
Supplement: Document S1. Figures S1–S4 and Table S1 [file mmc1.pdf]

Cell Reports, Volume *25*

Supplemental Information

BET Inhibition Induces HEXIM1- and RAD51-Dependent Conflicts between Transcription and Replication

Akhil Bowry, Ann Liza Piberger, Patricia Rojas, Marco Saponaro, and Eva Petermann

Supplemental Figures

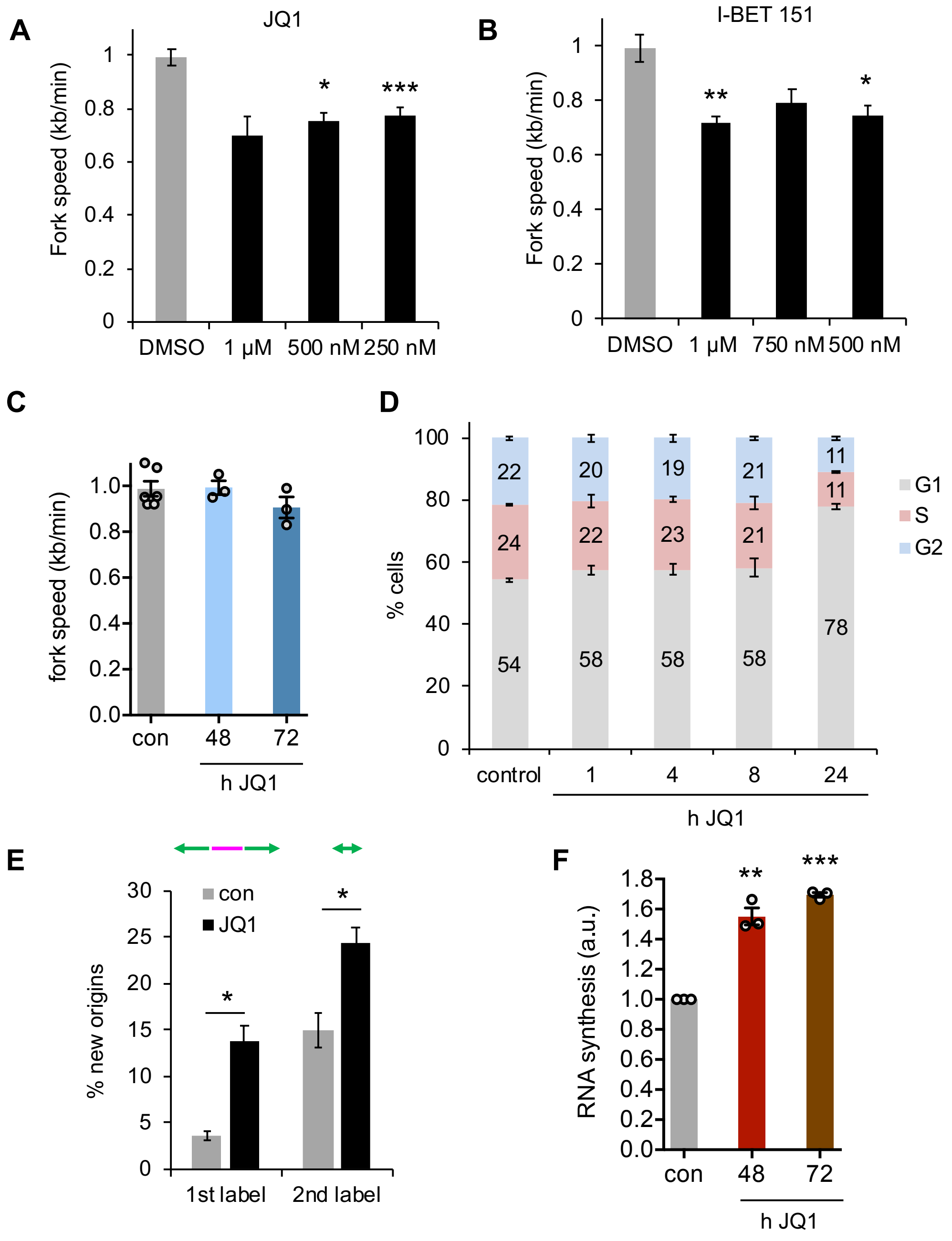

Figure S1. Related to Figure 1. BET inhibition induces replication fork slowing. A) Replication fork speeds in U2OS cells after treatment with 0.25-1 µM JQ1 for 1 h. n=3. B) Replication fork speeds in U2OS cells after treatment with 0.5-1 µM I-BET151 for 1 h. n=4. C) Replication fork speeds in U2OS cells treated with 1 µM JQ1 for 48 and 72 h. n=3. D) Cell cycle distribution measured by flow cytometry of U2OS cells after 1-24 h treatment with 1 µM JQ1. n=3. E) New origin firing in U2OS cells after treatment with 1 µM JQ1 for 1 h. n=3. F) Nuclear EU intensity in U2OS cells treated with 1 µM JQ1 for 48 and 72 h. n=3. Data are represented as mean ± SEM.

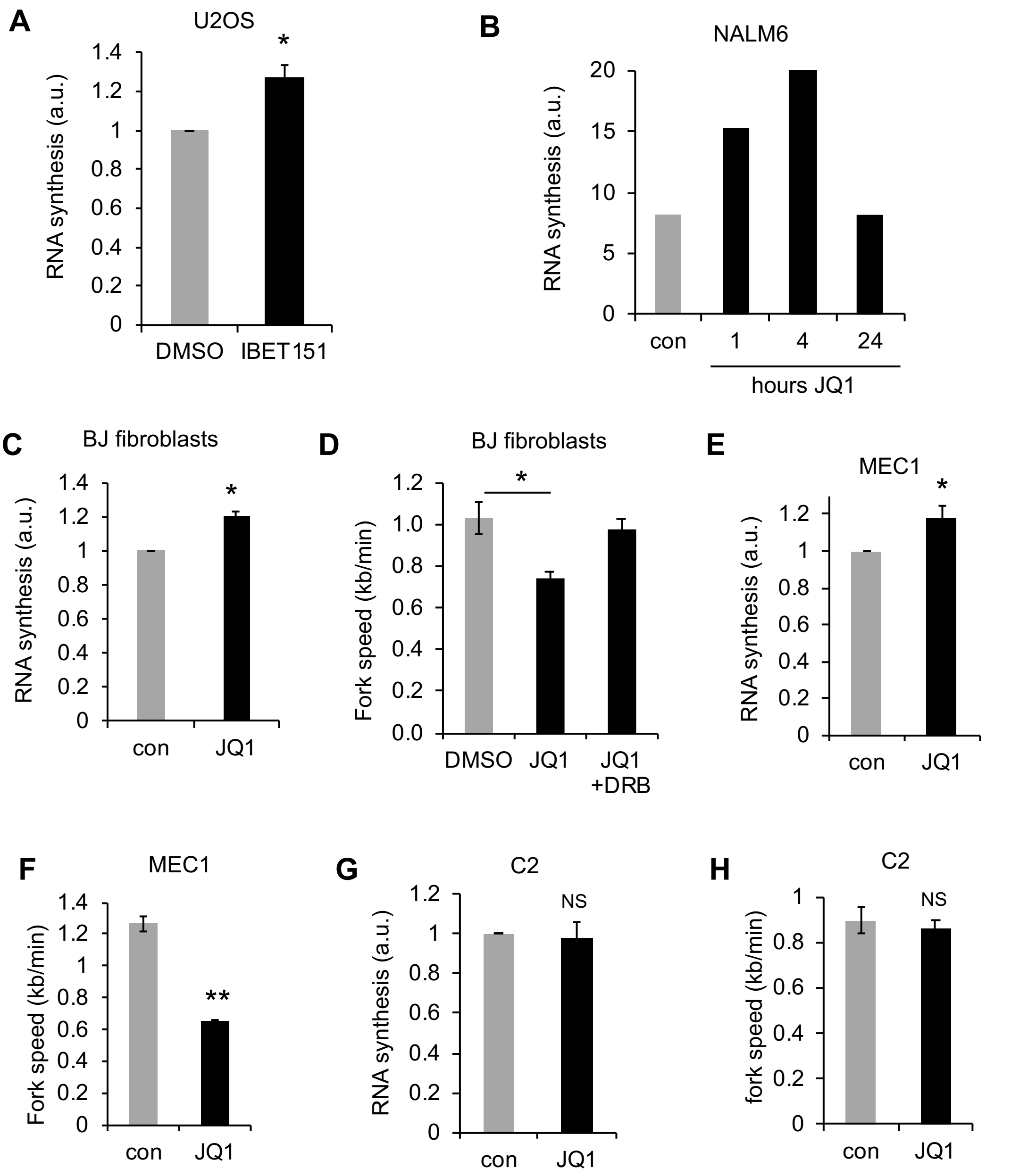

Figure S2. Related to Figure 1. Replication-transcription conflicts induced by I-BET151 and in a number of cell lines. A) Quantification of nuclear EU intensity in U2OS cells after 1 µM I-BET151 (1h). n=4. B) Nuclear EU intensity in NALM6 cells after 1-24h 1 µM JQ1. n=1. C) Nuclear EU intensity in BJ-hTert fibroblasts +/- 1 µM JQ1 (1h). n=3. D) Median replication fork speeds in BJ-hTert fibroblasts +/- 1 µM JQ1 (1h), and treated with JQ1 and DRB for 1h. n=3. E) Nuclear EU intensity in MEC1 cells +/-1 µM JQ1 (1h). n=3. F) Median replication fork speeds in MEC1 cells +/- 1 µM JQ1 (1h). n=5. G) Nuclear EU intensity in C2 cells +/-1 µM JQ1 (1h). n=3. H) Median replication fork speeds in C2 cells +/- 1 µM JQ1 (1h). n=3. Data are represented as mean ± SEM.

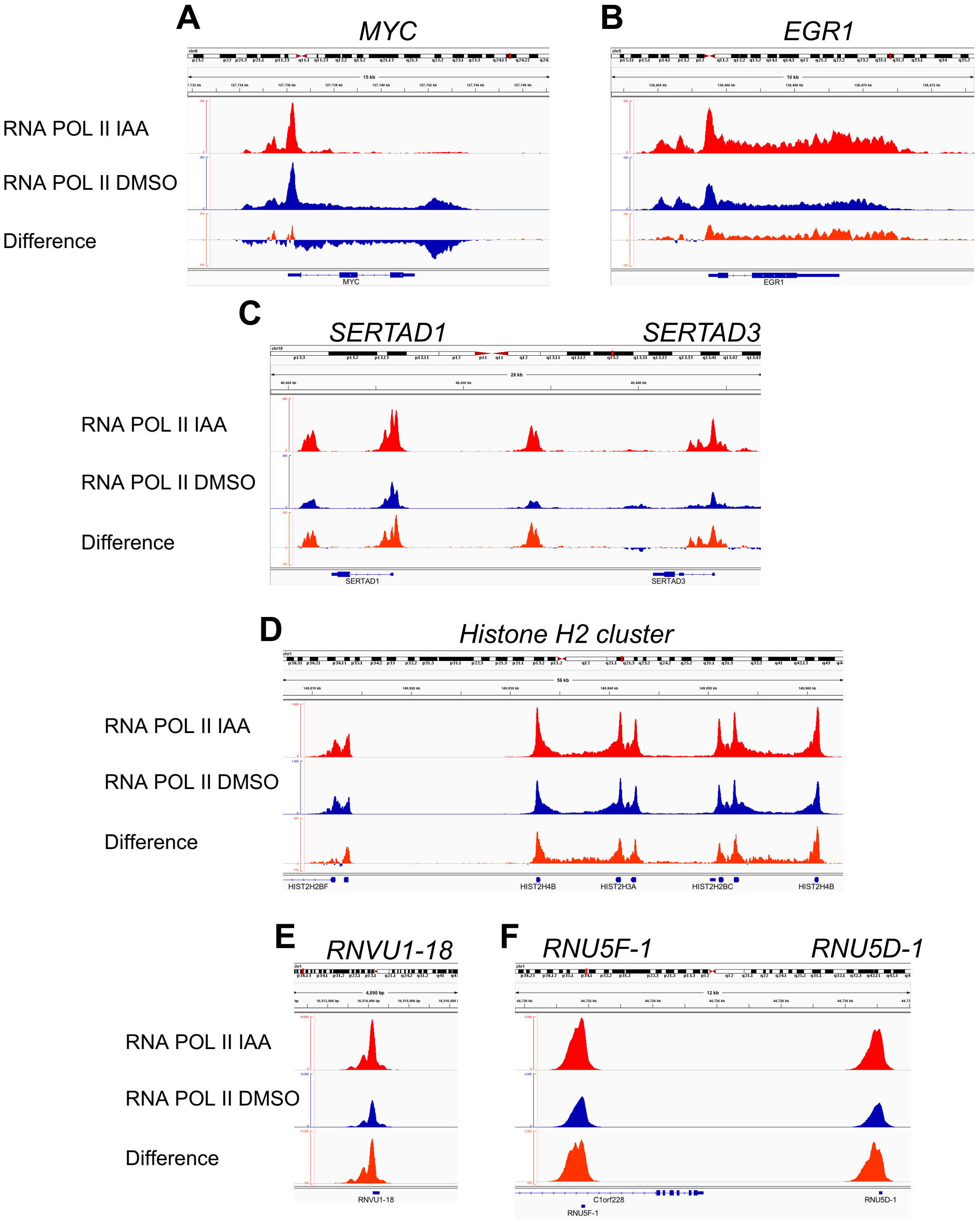

Figure S3. Related to Figure 1. BRD4 degradation increases RNA Pol II occupancy in histone and non-coding RNA genes. Analysis of published RNA Pol II ChIP-seq data sets (Muhar et al., 2018; GEO Series GSE111463). RNA Pol II ChIP was performed 1h after BRD4 degradation using auxin inducible degron (AID) approach in K562 leukaemia cells. The mRNA expression of *MYC*, *EGR1* and *SERTAD1* was analysed in parallel (Muhar et al., 2018). A) *MYC* (mRNA decreased 1h after BRD4 degradation), B) *EGR1* (mRNA increased 1h after BRD4 degradation), C) *SERTAD1* (mRNA increased 1h after BRD4 degradation), D) histone gene cluster (non-polyadenylated transcripts), also with large increase in RNA Pol II levels outside genes boundaries, E) non-coding RNA *RNVU1-18* (non-polyadenylated transcript), F) non-coding RNAs *RNU5F-1* and *RNU5D-1* (non-polyadenylated transcripts). Data are represented as mean ± SEM.

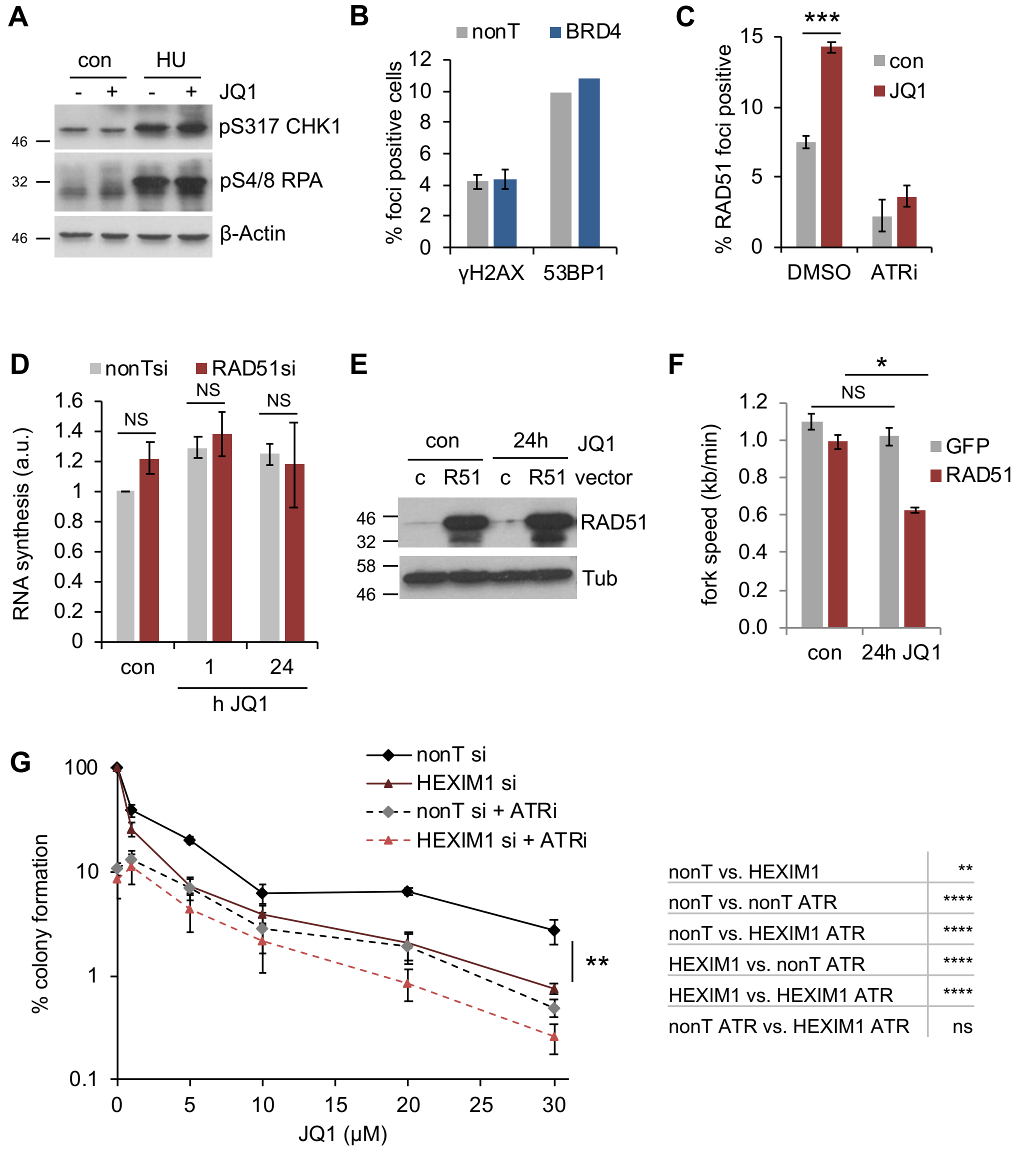

Figure S4. Related to Figure 4. DNA damage response to BET inhibition. A) Levels of phospho-S4/8 RPA32, phospho-S317 CHK1 and β-Actin after treatment with JQ1 and HU as indicated. B) Percentages of cells containing more than 8 γH2AX or 53BP1 foci after BRD4 depletion. n = 2 (γH2AX), n = 1 (53BP1). C) Percentages of cells containing more than 8 RAD51 foci after JQ1 treatment +/- ATR inhibitor AZ20. n=3 (DMSO), n=2 (ATRi). D) Quantification of nuclear EU intensity +/- RAD51 siRNA and JQ1 treatment. n=3. E) Levels of RAD51 and loading control after transient overexpression of RAD51 or eGFP (control) for 24 h +/- JQ1. F) Replication fork speeds after transient overexpression of RAD51 or eGFP (control) for 24 h +/- JQ1. n=3. G) Colony survival of U2OS cells treated with indicated concentrations of JQ1 for 24h, +/- HEXIM1 siRNA and AZ20. n=3. The statistical test used was 2-way ANOVA with Tukey's. Data are represented as mean ± SEM.

Supplementary Table S1. Related to STAR Methods. Sequences of PCR primers

Name	Sequence (5′ → 3′)
HIST2H3D For	CGAGATCGCGCAGGACTTTA
HIST2H3D Rev	TGTCCTTGGGCATGATGGTC
HIST2H4B For	GAAGCTGTCTATCGGGCTCC
HIST2H4B Rev	ACTGCGTCCCGAATCACATT
HIST2H2BE For	GCCACCCACCTAATCACTAGAAA
HIST2H2BE Rev	CAATGACGCACTGGGGACC
HIST4H4 For	GGGTCGCACCCTTTATGGTT
HIST4H4 Rev	GTATCCGGAAGGCGACATCA
EGR1 For	TGCAGATCTCTGACCCGTTC
EGR1 Rev	CAGGAAAAGACTCTGCGGTCA
SERTAD1 For	GAGGACAGCCAACAAGCGAT
SERTAD1 Rev	TGCTCAGCATCTTGCTCACTA
MYC For	CAGCGACTCTGAGGAGGAAC
MYC Rev	GCTGCGTAGTTGTGCTGATG
RNU5D1 For	GCTCTGGTTTCTCTTCAAAT
RNU5D1 Rev	AACCCCACCAACATAGGG
RNVU1-18 For	GGTTTTCCCAGGGCGAGG
RNVU1-18 Rev	CCACTACCACAAATTATGCAGTC
RPLP0 F2 (control)	CAGATTGGCTACCCAACTGTT
RPLP0 R2 (control)	GGAAGGTGTAATCCGTCTCCAC
