# Supplementary material for: BET Inhibition Induces HEXIM1- and RAD51-Dependent Conflicts between Transcription and Replication
Source: Cell Rep. 2018 Nov 20;25(8):2061–2069.e4. doi: 10.1016/j.celrep.2018.10.079 (PMC6280123; doi:10.1016/j.celrep.2018.10.079)
Supplement: Document S2. Article plus Supplemental Information [file mmc2.pdf]

Cell Reports

Report

BET Inhibition Induces HEXIM1- and RAD51-Dependent Conflicts between Transcription and Replication

Graphical Abstract

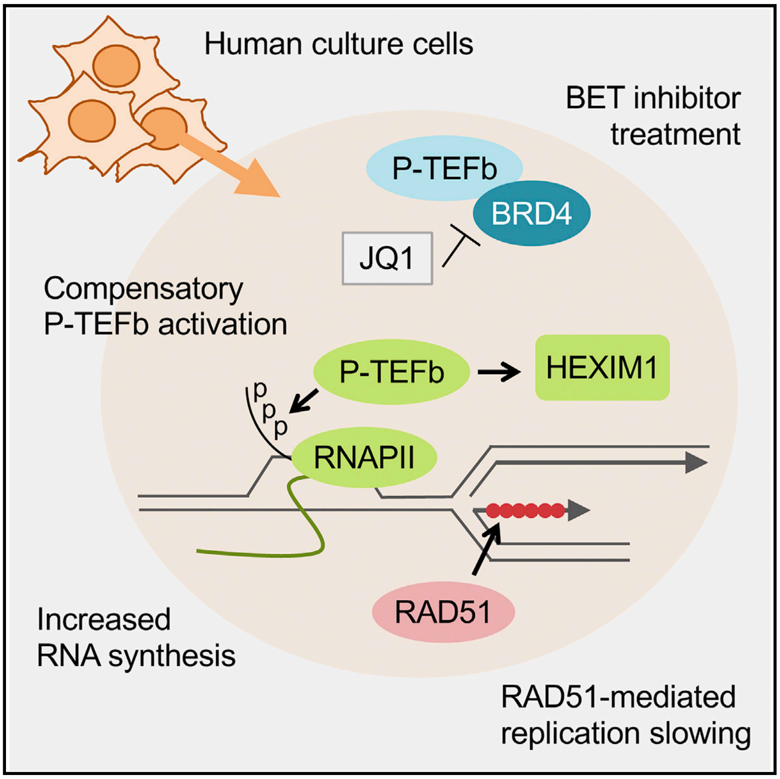

Highlights

- BET inhibitors and BRD4 depletion increase overall RNA synthesis
- Loss of BRD4 activity causes conflicts between transcription and DNA replication
- These transcription-replication conflicts depend on P-TEFb inhibitor HEXIM1
- BET inhibition activates RAD51 to slow replication forks and suppress DNA damage

Authors

Akhil Bowry, Ann Liza Piberger, Patricia Rojas, Marco Saponaro, Eva Petermann

Correspondence

e.petermann@bham.ac.uk

In Brief

Bowry et al. show that BET inhibitors, emerging cancer therapeutics that target transcription programs, cause conflicts between transcription and replication by deregulating P-TEFb. These conflicts recruit the homologous recombination factor RAD51, which slows down replication and suppresses DNA damage. This highlights the importance of replication stress for BET inhibitor treatment.

Bowry et al., 2018, Cell Reports *25*, 2061–2069
November 20, 2018 © 2018 The Author(s).
https://doi.org/10.1016/j.celrep.2018.10.079

OPEN ACCESS
CellPress

BET Inhibition Induces HEXIM1- and RAD51-Dependent Conflicts between Transcription and Replication

Akhil Bowry,[1] Ann Liza Piberger,[1] Patricia Rojas,[1] Marco Saponaro,[1] and Eva Petermann[1,2,*]
[1]Institute of Cancer and Genomic Sciences, College of Medical and Dental Sciences, University of Birmingham, Birmingham B15 2TT, UK
[2]Lead Contact
*Correspondence: e.petermann@bham.ac.uk
https://doi.org/10.1016/j.celrep.2018.10.079

SUMMARY

BET bromodomain proteins are required for oncogenic transcription activities, and BET inhibitors have been rapidly advanced into clinical trials. Understanding the effects of BET inhibition on processes such as DNA replication will be important for future clinical applications. Here, we show that BET inhibition, and specifically inhibition of BRD4, causes replication stress through a rapid overall increase in RNA synthesis. We provide evidence that BET inhibition acts by releasing P-TEFb from its inhibitor HEXIM1, promoting interference between transcription and replication. Unusually, these transcription-replication conflicts do not activate the ATM/ATR-dependent DNA damage response but recruit the homologous recombination factor RAD51. Both HEXIM1 and RAD51 promote BET inhibitor-induced fork slowing but also prevent a DNA damage response. Our data suggest that BET inhibitors slow replication through concerted action of transcription and recombination machineries and shed light on the importance of replication stress in the action of this class of experimental cancer drugs.

INTRODUCTION

Members of the BET bromodomain-containing protein family bind to lysine-acetylated histone tails and regulate transcription by recruiting and activating positive transcription elongation factor b (P-TEFb). P-TEFb can occur in two active complexes with BET protein BRD4 or the super elongation complex (SEC) and an inactive complex with 7SK-snRP (7SK RNA, HEXIM1, LARP7, and MEPCE). BRD4 activates P-TEFb by releasing it from 7SK-snRP and recruits active P-TEFb to gene promoters (Quaresma et al., 2016). Active P-TEFb facilitates RNA polymerase II (Pol II) pause release by phosphorylating the RNA Pol II C-terminal domain (CTD) and other targets.

BET proteins promote oncogenic transcription programs, and specific small-molecule inhibitors of BET bromodomains promise a targeted cancer treatment (Delmore et al., 2011). BET inhibition downregulates MYC protein levels and kills tumor cells independently of p53 (Da Costa et al., 2013). In solid tumor cells, BET inhibitor responses can be *MYC* independent (Lockwood et al., 2012). Although the molecular mechanisms surrounding BET inhibitor action are still poorly understood, BET inhibitors are already undergoing clinical trials in a wide range of cancers (Andrieu et al., 2016; Fujisawa and Filippakopoulos, 2017).

More recently, BRD2 and BRD4 have been implicated in DNA replication and DNA damage responses (Da Costa et al., 2013; Floyd et al., 2013; Sansam et al., 2018). BRD4 in particular interacts with DNA replication factors RFC, TICRR, and CDC6 (Maruyama et al., 2002; Sansam et al., 2018; Zhang et al., 2018). Inhibiting the interaction between BRD2/4 and TICRR slowed euchromatin replication, suggesting that BET proteins control DNA replication initiation to prevent interference between replication and transcription (Sansam et al., 2018). BET inhibitors cause little or no DNA damage but promote downregulation of DNA replication stress-response and stress-repair genes (Pawar et al., 2018; Zhang et al., 2018). It is not known whether the latter are specific responses to BET inhibition affecting replication and repair. Investigating more direct effects of BET proteins and BET inhibition on DNA replication might help understand BET inhibitor action independently of cell-type-specific transcription programs and provide insights into potential side effects and resistance mechanisms.

We previously reported that JQ1 treatment slows replication fork progression in NALM-6 leukemia cells, indicative of replication stress (Da Costa et al., 2013). Replication stress occurs when the transcription machinery or other obstacles hinder replication fork progression, which promotes formation of mutagenic or cytotoxic DNA damage, especially double-strand breaks (DSBs). This is highly relevant to cancer therapy, as many conventional chemotherapies act by causing severe replication stress and collapse of replication forks into DSBs. However, non-toxic levels of replication stress can promote genomic instability, an unwanted side effect of cancer therapy (Kotsantis et al., 2015).

Here we describe a mechanism by which BET inhibition causes replication stress. We show that BET inhibition and loss of BRD4 cause rapid upregulation of RNA synthesis and transcription-dependent replication fork slowing in a pathway that depends on HEXIM1 and RAD51. Unexpectedly, combination of BET inhibitor with HEXIM1 or RAD51 depletion prevents fork slowing but activates a DNA damage response, suggesting that replication fork slowing might help suppress BET inhibitor-induced DNA damage.

This is an open access article under the CC BY license (http://creativecommons.org/licenses/by/4.0/).

RESULTS

U2OS osteosarcoma cells were used as a well-characterized model for replication stress and DNA damage. Osteosarcoma is one of many cancers proposed to benefit from BET inhibitor treatment (Lamoureux et al., 2014). We confirmed that JQ1 treatment slowed replication within 1 hr (Figures 1A and 1B). Replication was also slowed by lower concentrations of JQ1 and another BET inhibitor, I-BET151 (Figures S1A and S1B).

As reported previously (Da Costa et al., 2013), replication forks speeds were recovered to control levels after 24 hr incubation with JQ1 and remained at control levels for up to 72 hr (Figure S1C). This was not due to loss of JQ1 activity, because adding fresh JQ1 after 23 hr did not slow fork speeds (Figures 1A and 1B). This suggests that replication forks are rapidly slowed by JQ1 treatment, but they eventually adapt. Cell cycle distribution remained unaffected between 1 and 8 hr JQ1 treatment, but cells accumulated in G1 after 24 hr JQ1 treatment (Figure S1D). The lack of S-phase arrest could be explained by compensatory new origin firing (Figure S1E).

To investigate whether ongoing transcription contributes to JQ1-induced replication slowing, we quantified nascent RNA synthesis using nuclear incorporation of 5-ethynyluridine (EU) (Figure 1C). EU incorporation increased by about 35% after 1 hr JQ1 treatment and remained increased up to 72 hr of JQ1 treatment (Figures 1D, 1E, and S1F). Increased RNA synthesis was also observed in U2OS cells treated with I-BET151 and in NALM-6 cells (Figures S2A and S2B). For an alternative approach, we isolated total RNA and normalized yields to cell numbers, showing that JQ1-treated cells contained more RNA overall (Figure 1F).

In contrast to our findings, it was previously reported that JQ1 treatment or BRD4 degradation quickly suppress nascent transcription of most protein-coding genes such as *MYC*, with only a few genes such as *EGR1* and *SERTAD1* upregulated (Muhar et al., 2018; Winter et al., 2017). As previous work focused on poly-adenylated mRNA sequencing, we decided to investigate the effect of BET inhibition on non-poly-adenylated RNA species. First, we re-analyzed the published RNA Pol II chromatin immunoprecipitation sequencing (ChIP-seq) datasets (Muhar et al., 2018). These showed that 1 hr after BRD4 degradation, genome-wide net occupancy of RNA Pol II actually increased by 53.8%, particularly over a set of highly transcribed genes that produce non-poly-adenylated RNAs such as histone and non-coding RNA genes (Figure S3). We used qRT-PCR to test whether this increased RNA Pol II occupancy was also increasing gene expression. Indeed, expression of all selected candidate genes was also upregulated by JQ1 in U2OS cells (Figure 1G). Our data support that although BET inhibition suppresses transcription of poly-adenylated protein-coding genes, highly transcribed histone and other non-poly-adenylated non-coding RNA genes are upregulated, and this may explain the observed increase in total nascent RNA synthesis.

To test whether replication fork slowing was transcription dependent, we used short treatments with the transcription inhibitors triptolide, DRB, and α-amanitin (Figure 1H). These inhibited ongoing RNA synthesis and increased replication fork speeds specifically in the presence of JQ1 (Figures 1I and 1J). Similar results were observed in human non-cancer BJ-hTert fibroblasts and in two chronic leukemia cell lines, C2 and MEC1 (Figures S2C–S2H). These data suggest that JQ1-induced replication stress depends on active RNA synthesis and that this is not restricted to cancer cells. One hour JQ1 treatment increased RNA synthesis in four of five cell lines tested, which was always accompanied by fork slowing (Figure 1K). Fork slowing was more dramatic in leukemia lines compared with U2OS or fibroblasts. Only C2 cells appeared resistant to JQ1 effects, displaying neither increased RNA synthesis nor fork slowing.

We used small interfering RNA (siRNA) to investigate which BET protein was the target of JQ1-induced replication-transcription conflicts. We first depleted BRD4, which can interact with DNA replication proteins (Maruyama et al., 2002; Sansam et al., 2018; Zhang et al., 2018). BRD4 depletion increased RNA synthesis (Figures 2A–2D) and reduced fork speeds (Figure 2E). Adding JQ1 did not further affect fork speeds in BRD4-depleted cells (Figure 2E). BRD4 siRNA-induced fork slowing was rescued by ectopic expression of the long isoform of BRD4 (Figures 2F and 2G) and short transcription inhibitor treatments (Figure 2H). In contrast, depletion of BRD2 or BRD3 did not increase RNA synthesis and caused negligible fork slowing (Figures 2I–2N). These data suggest that BRD4 is the BET protein that prevents replication-transcription conflicts and is required for BET inhibitor-induced replication stress.

We next investigated the mechanism of increased RNA synthesis. It has been shown that both JQ1 and I-BET151 can disrupt the 7SK-snRP-P-TEFb complex, increasing the proportion of active P-TEFb in complex with the SEC (Bartholomeeusen et al., 2012; Chaidos et al., 2014). This promotes transcription of genes including the 7SK-snRP component *HEXIM1* (Bartholomeeusen et al., 2012). Increased HEXIM1 protein levels eventually re-establish the 7SK-snRP-pTEFB complex and therefore P-TEFb inhibition (Figure 3A). We hypothesized that JQ1-induced replication stress might result from 7SK-snRP dissociation and increased RNA Pol II activity. In line with this, RNA Pol II CTD serine2 phosphorylation was transiently increased during the first hours of JQ1 treatment (Figures 3B and 3C). Several drugs including hexamethylene bis-acetamide (HMBA) can dissociate 7SK-snRP from P-TEFb (Fujinaga et al., 2015). If 7SK-snRP dissociation underlies BET inhibitor-induced fork slowing, then HMBA treatment should also slow replication, even though HMBA has no known connection to replication forks. HMBA treatment slightly increased RNA synthesis and slowed replication forks, which was not further exacerbated by co-treatment with JQ1 (Figures 3D and 3E). Finally, the short isoform of BRD4, which cannot effectively interact with P-TEFb (Schröder et al., 2012), failed to rescue the effect of BRD4 knockdown on replication fork slowing (Figures 3F–3H).

We decided to further investigate the roles of HEXIM1 in JQ1-induced RNA synthesis and replication fork slowing. HEXIM1 depletion prevented the early JQ1-induced increase in RNA synthesis and replication fork slowing. After 24 hr JQ1 treatment, however, RNA synthesis increased, accompanied by fork slowing (Figures 3I–3L). These data suggest that HEXIM1 depletion delayed the effects of JQ1. Although HEXIM1 protein levels slowly increased during 48–72 hr JQ1 treatment, there was no decrease in nascent RNA synthesis (Figures 3J and S1F),

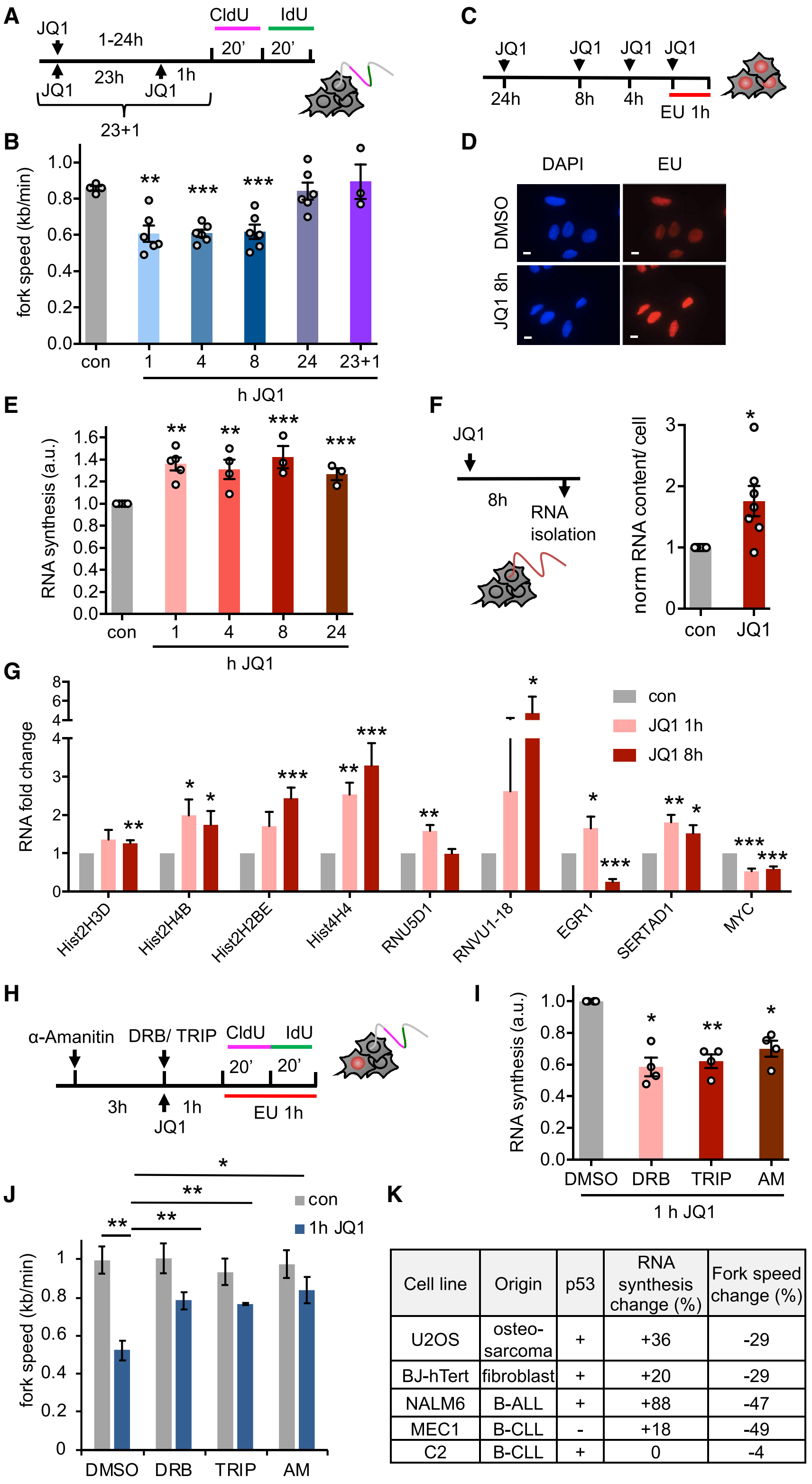

Cell line	Origin	p53	RNA synthesis change (%)	Fork speed change (%)
U2OS	osteo-sarcoma	+	+36	-29
BJ-hTert	fibroblast	+	+20	-29
NALM6	B-ALL	+	+88	-47
MEC1	B-CLL	-	+18	-49
C2	B-CLL	+	0	-4

Figure 1. BET Inhibition Induces Replication-Transcription Conflicts

(A) DNA fiber labeling in U2OS cells treated with JQ1.

(B) Replication fork speeds after JQ1 treatment (n = 3–6).

(C) EU labeling after JQ1 treatment.

(D) Representative images of click-stained EU labeled cells ± 8 hr JQ1.

(E) Nuclear EU intensities after JQ1 treatment (n = 3–5).

(F) RNA was extracted after 8 hr JQ1 treatment and yield normalized to cell number and DMSO (n = 7).

(G) Fold change in the normalized expression levels of indicated transcripts ± JQ1 as indicated (n = 4).

(H) Cells were treated with transcription inhibitors before and during EU or DNA fiber labeling. AM, α-amanitin; TRIP, triptolide.

(I) Nuclear EU intensities in cells treated with transcription inhibitors and JQ1 (n = 4).

(J) Replication fork speeds after 1 hr JQ1 ± transcription inhibitors (n = 3 or 4).

(K) JQ1 effect on nascent RNA synthesis and replication fork speeds in a panel of human cell lines.

Data are represented as mean ± SEM. Scale bars, 10 μm. See also Figures S1–S3 and Table S1.

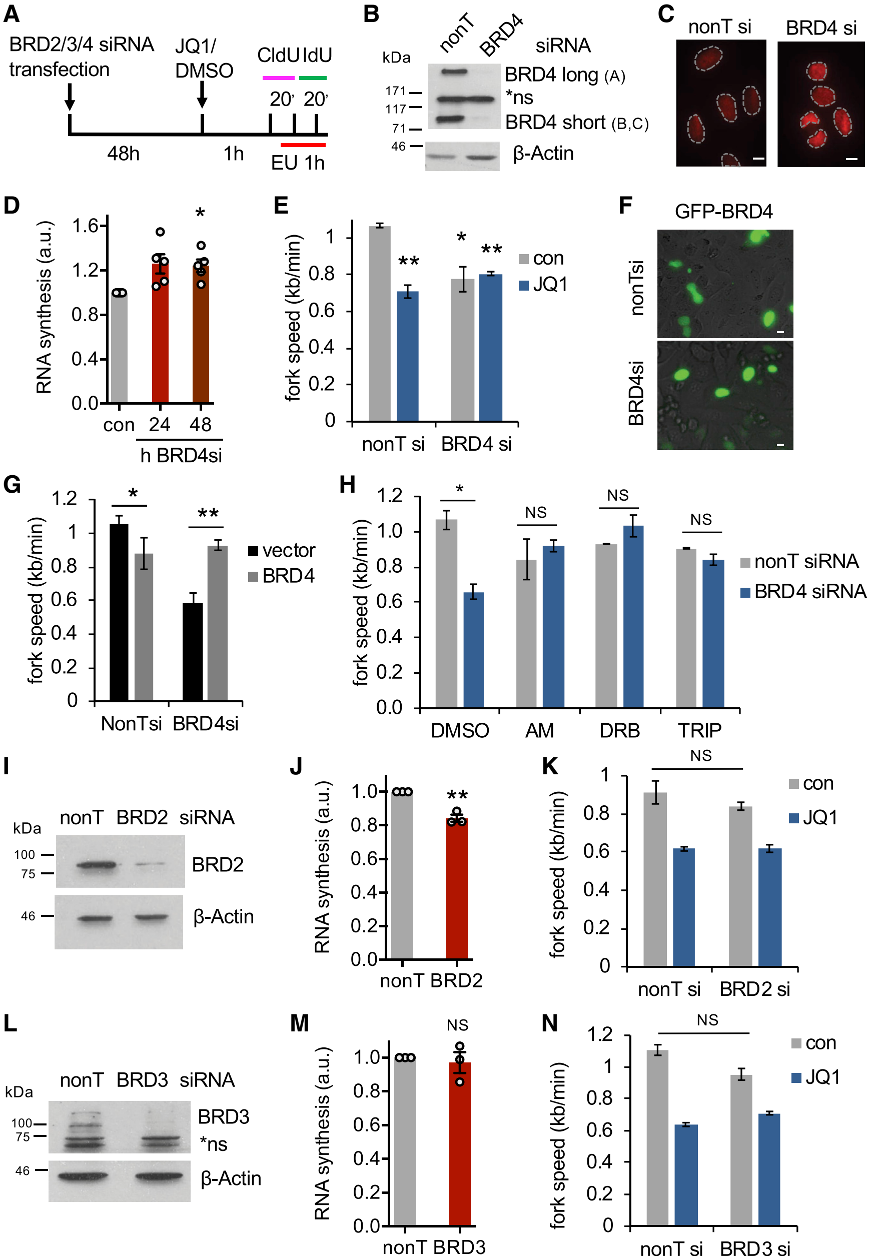

Figure 2. Loss of BRD4 Causes Replication-Transcription Conflicts

(A) DNA fiber labeling after BRD2/3/4 depletion.
(B) Protein levels of BRD4 isoforms after siRNA depletion. NS, non-specific bands.
(C) Representative EU images ± BRD4 siRNA.
(D) Nuclear EU intensities ± BRD4 siRNA (n = 5).
(E) Replication fork speeds after BRD4 depletion ± 1 hr JQ1 (n = 3).
(F) Equal EmGFP-BRD4 expression 48 hr after plasmid transfection ± BRD4 siRNA.
(G) Replication fork speeds after BRD4 siRNA ± BRD4 long isoform expression plasmid (n = 3).
(H) Median replication fork speeds after BRD4 siRNA ± transcription inhibitors (n = 3).
(I) Protein levels of BRD2 after siRNA depletion.
(J) Nuclear EU intensities ± BRD2 siRNA (n = 3).
(K) Replication fork speeds after BRD2 siRNA ± 1 hr JQ1 (n = 3).
(L) Protein levels of BRD3 after siRNA depletion. NS, non-specific.
(M) Nuclear EU intensities ± BRD3 siRNA (n = 3).
(N) Replication fork speeds after BRD3 siRNA ± 1 hr JQ1 (n = 3).
Data are represented as mean ± SEM. Scale bars, 10 μm.

suggesting that the process of replication adaptation is more complex than a HEXIM1-mediated feedback loop suppressing transcription.

We then investigated the relationship between JQ1-induced fork slowing and DNA damage. Replication fork slowing can expose single-stranded DNA (ssDNA) and, if forks collapse, cause DSBs. These activate the ATR and ATM checkpoint kinases and p53. However, JQ1 does not induce DNA damage (Pawar et al., 2018) or activate p53 (Da Costa et al., 2013). In line with this, we observed no JQ1-induced increase in ssDNA or DSBs as measured by nuclear foci formation of RPA and 53BP1 or phosphorylation of the ATM and ATR targets histone H2AX (γH2AX; Figure 4A), CHK1, and RPA (Figure S4A). BRD4 depletion also failed to induce DNA damage foci (Figure S4B).

Unexpectedly however, JQ1 induced foci formation of the homologous recombination factor RAD51, which depended on ongoing transcription and ATR activity (Figures 4B–4D and S4C). This suggested that RAD51 is recruited in response to JQ1-induced transcription-replication conflicts, aided by basal ATR activity. We used siRNA to investigate the impact of RAD51 on replication fork progression in JQ1-treated cells (Figures 4E and 4F). Interestingly, RAD51 depletion prevented JQ1-induced fork slowing (Figure 4G). We investigated whether RAD51 suppresses RNA synthesis, like HEXIM1. RAD51-depleted cells displayed high levels of RNA synthesis in both the presence and absence of JQ1, making these data difficult to interpret (Figure S4D). Nevertheless, we concluded that RAD51-dependent rescue of fork speeds was not due to decreased transcription. Transient overexpression of RAD51 promoted fork slowing after 24 hr JQ1 treatment, additionally supporting that RAD51 directly slows forks in response to JQ1 (Figures S4E and S4F).

RAD51-mediated fork slowing was previously reported for DNA-damaging treatments and is suggestive of replication fork reversal (Zellweger et al., 2015). In support of a fork reversal model, PARP inhibition and depletion of SMARCAL1 or ZRANB3 (Bhat and Cortez, 2018) also prevented JQ1-induced fork slowing (Figure 4H-J).

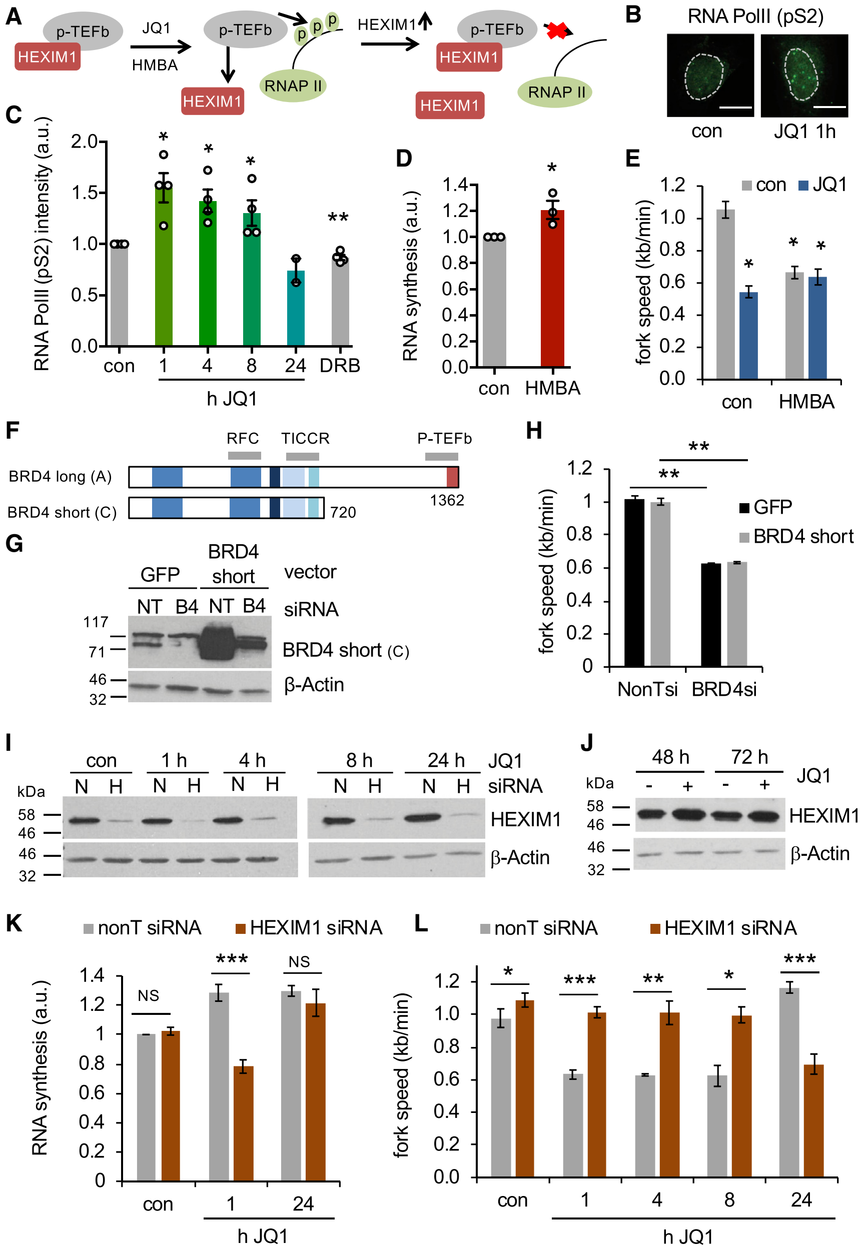

Figure 3. BET Inhibitor-Induced Replication-Transcription Conflicts Require HEXIM1

(A) Current model HEXIM1 role in JQ1-induced transcription increase.

(B) Nuclear immunostaining for phospho-S2 RNA Pol II ± JQ1.

(C) Nuclear phospho-S2 RNA Pol II intensities as in (B) (n = 4; n = 2 [24 hr]).

(D) Nuclear EU intensities ± 5 mM HMBA (1 hr) (n = 3).

(E) Replication fork speeds after HMBA treatment ± JQ1 (1 hr) (n = 3).

(F) Schematic of BRD4 isoforms used.

(G) Protein levels of BRD4 short isoform after full-length BRD4 siRNA ± BRD4 short isoform expression plasmid.

(H) Replication fork speeds after full-length BRD4 siRNA ± BRD4 short isoform expression plasmid (n = 3).

(I) Protein levels of HEXIM1 after 48 hr siRNA transfection followed by JQ1 for times indicated.

(J) Protein levels of HEXIM1 after 48–72 hr JQ1.

(K) Nuclear EU intensities after HEXIM1 siRNA and JQ1 treatment (n = 3–9).

(L) Replication fork speeds after HEXIM1 siRNA and JQ1 treatment (n = 3 or 4, n = 2 [nonT 8 hr]).

Data are represented as mean ± SEM. Scale bars, 10 μm.

RAD51, which acts downstream of these conflicts, contribute to replication fork slowing and prevent JQ1-induced DNA damage (Figure 4P).

DISCUSSION

We report that BET inhibition increases transcription especially of highly transcribed histone and other non-poly-adenylated non-coding RNA genes and causes transcription-dependent replication fork slowing. This depends on HEXIM1, a central factor in the BET inhibitor response, and the homologous recombination factor RAD51, which is central in the replication stress response. Unusually, BET inhibitor-induced replication stress is transient and, despite engaging RAD51, does not activate a full DNA damage response. We speculate that these unusual transcription-replication conflicts will help illuminate some aspects of BET inhibitor treatment responses.

Our data provide insight into the roles of BRD4 in DNA replication. Reports show that BRD4 interacts with DNA replication factors RFC, TICRR, and CDC6 (Maruyama et al., 2002; Sansam et al., 2018; Zhang et al., 2018). We show here that BRD4 also regulates DNA replication via its P-TEFb interaction. The BRD4 short isoform contains the interaction domains for TICRR and RFC (Maruyama et al., 2002; Sansam et al., 2018) but failed to rescue replication stress. The interaction with CDC6 is not yet

We then tested the effect of RAD51 depletion on JQ1-induced DNA damage. Interestingly, RAD51 depletion actually promoted DNA damage, as indicated by γH2AX and 53BP1 foci formation (Figures 4K–4M). This suggests that RAD51 prevents DNA damage at JQ1-slowed replication forks.

As HEXIM1 depletion also rescued fork progression, we tested the effect of HEXIM1 on DNA damage. HEXIM1-depleted cells accumulated DNA damage early during JQ1 treatment that persisted for 24 hr (Figures 4N and 4O). In line with the increased DNA damage, HEXIM1-depleted cells were more sensitive to 24 hr JQ1 treatment than control cells, as were ATR inhibitor-treated cells (Figure S4G). This suggests that both HEXIM1, which is upstream of transcription-replication conflicts, and

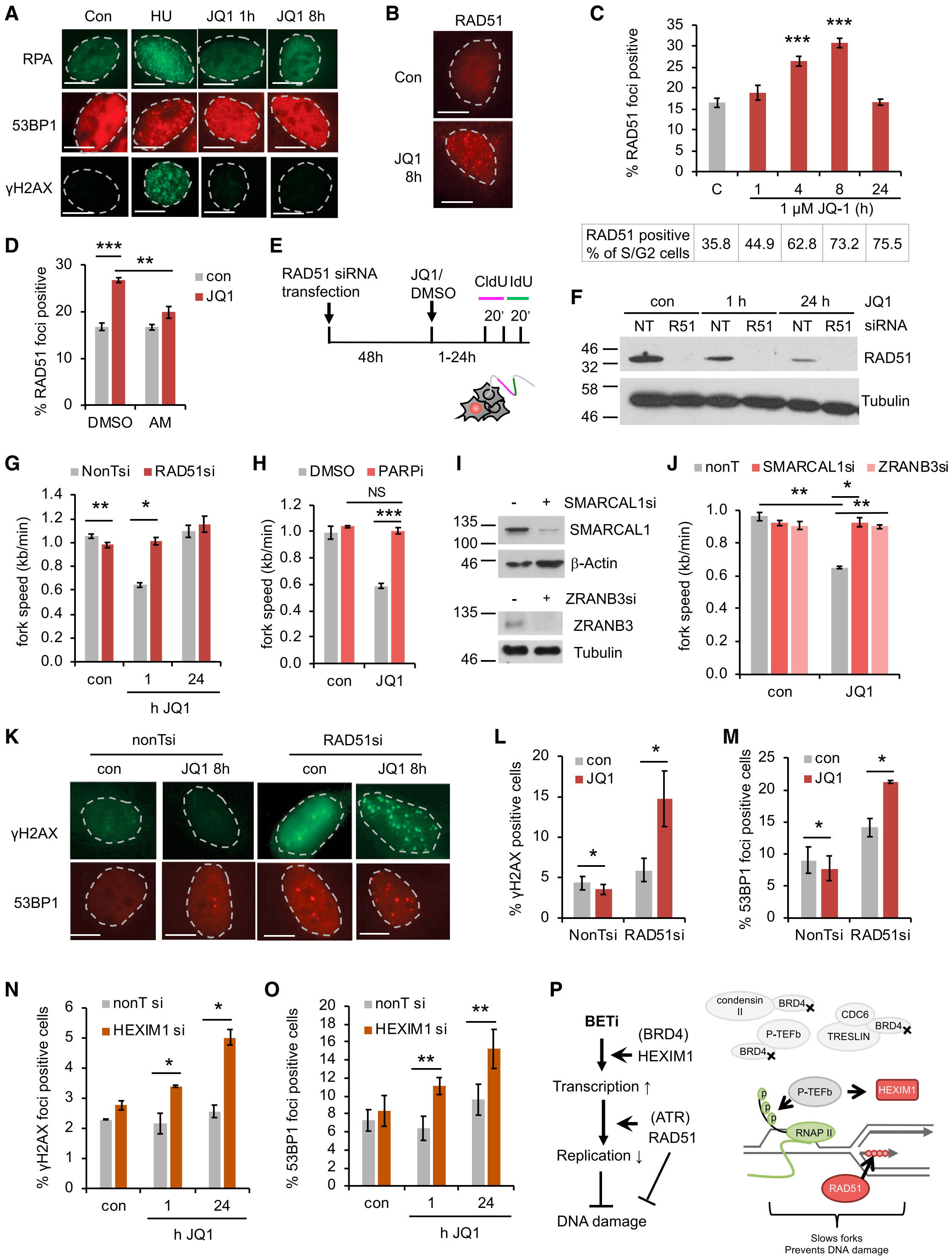

Figure 4. RAD51 and HEXIM1 Modulate BET Inhibitor-Induced DNA Damage

(A) Representative images of DNA damage foci after JQ1 treatment. HU was 2 mM 8 hr.

(B) Representative images of RAD51 foci after JQ1 treatment.

(C) Total percentages (top) and percentage normalized to S/G2 content (bottom) of cells with RAD51 foci after JQ1 treatment (n = 3–6).

(D) Percentages of cells with RAD51 foci ± 4 hr JQ1 and α-amanitin (AM) (n = 3).

(E) EU and DNA fiber labeling after RAD51 depletion.

(legend continued on next page)

mapped (Zhang et al., 2018). Our data suggest that TICRR and RFC are not involved in the phenotypes described here. Instead, they support previous reports that BET inhibition rapidly increases P-TEFb activity (Bartholomeeusen et al., 2012; Chaidos et al., 2014). We show that this also involves increased RNA synthesis. Our data support that although BET inhibition suppresses transcription of poly-adenylated protein-coding genes (Muhar et al., 2018; Winter et al., 2017), non-poly-adenylated RNA Pol II transcripts such as histones and non-coding RNA genes are upregulated. We previously reported that overexpression of H-RAS^{V12} or transcription factor TBP increases nascent RNA synthesis and causes replication stress (Kotsantis et al., 2016). BET inhibition shows how small-molecule inhibitor treatments can also cause replication stress by increasing RNA synthesis. Any treatments that disrupt the complex of P-TEFb with 7SK-snRP, including other cancer drugs, such as HDAC inhibitors and azacytidine (Fujinaga et al., 2015), could potentially cause transcription-replication conflicts in this way.

Our findings suggest that HEXIM1 is required for BET inhibitor-induced replication fork slowing, which is delayed by at least 8 hr in the absence of HEXIM1. After 24 hr JQ1, extensive gene expression changes and possibly HEXIM2 (Byers et al., 2005) may compensate for HEXIM1 loss. HEXIM1 depletion has been associated with long-term BET inhibitor resistance. Our findings do not conflict with this, as BET inhibitor resistance in HEXIM1-depleted cells was observed only after prolonged (>24 hr) treatment (Devaraj et al., 2016). It was proposed that HEXIM1 loss counteracts JQ1 by increasing P-TEFb activity (Devaraj et al., 2016). Importantly, we show that HEXIM1 loss can also promote JQ1 effects, such as DNA damage. In addition to modulating transcription-dependent fork slowing, HEXIM1 might play undiscovered roles in replication stress and DNA damage response. It may be relevant that HEXIM1 also regulates p53 (Lew et al., 2012). Nevertheless, we observed JQ1-induced replication fork slowing in a p53 mutant cell line. Reduced HEXIM1 protein levels have been observed in metastatic breast cancer (Ketchart et al., 2013), melanoma (Tan et al., 2016), and acute leukemia (Devaraj et al., 2016; Huang et al., 2016). It will be important for cancer researchers to further investigate the relationship between HEXIM1, replication stress, and the response to cancer treatment.

Homologous recombination capacity of cancer cells is a well-established predictive biomarker for the response to replication stress-inducing treatments. RAD51 loading is known to actively slow forks in response to a variety of genotoxic agents (Zellweger et al., 2015). We show that BET inhibition also activates RAD51, likely because of increased transcription, and that RAD51 promotes fork slowing in response to BET inhibitor, which may involve fork reversal. Forks may reverse at sites of direct collisions between replication and transcription machineries or in response to indirect effects of transcription on replication. RAD51 expression is downregulated in response to BET inhibition (Yang et al., 2017) and in models of acquired BET inhibitor resistance (Pawar et al., 2018). Intriguingly, acquired BET inhibitor resistance models also displayed increased DNA damage signaling (Pawar et al., 2018). This agrees with our data that RAD51 downregulation increases DNA damage signaling.

Our data support a speculative model whereby loss of HEXIM1 or RAD51 allows normal replication fork progression in the presence of BET inhibitor at the expense of DNA damage. This may even contribute to long-term BET inhibitor resistance. Although it is extensively documented that transcription-replication conflicts promote DNA damage, BET inhibition seems to induce DNA damage when such conflicts are prevented. This latter damage might, for example, result from reduced chromatin recruitment of chromatin remodelers (Floyd et al., 2013) or DNA replication proteins (Sansam et al., 2018; Zhang et al., 2018) (Figure 4P). The underlying mechanisms, and how they relate to BET inhibitor response, will require future investigation. In summary, we provide insights into the relationship among BET proteins, DNA replication, and DNA damage response that will be relevant to cancer therapy research.

STAR★METHODS

Detailed methods are provided in the online version of this paper and include the following:

- KEY RESOURCES TABLE
- CONTACT FOR REAGENT AND RESOURCE SHARING
- EXPERIMENTAL MODEL AND SUBJECT DETAILS
- METHOD DETAILS
  - Drug treatments
  - DNA fiber analysis
  - EU incorporation assay
  - siRNA and DNA transfection
  - Immunofluorescence
  - Western blotting
  - RNA isolation

(F) Protein levels of RAD51 after siRNA depletion.
(G) Replication fork speeds after 1 hr JQ1 ± RAD51 siRNA (n = 3 and 4).
(H) Replication fork speeds after 1 hr JQ1 ± PARP inhibitor (n = 3).
(I) Protein levels of SMARCAL1 or ZRANB3 48 hr after siRNA transfection.
(J) Replication fork speeds after 4 hr JQ1 ± SMARCAL1 or ZRANB3 siRNA (n = 3).
(K) Representative images of γH2AX and 53BP1 foci after JQ1 ± RAD51 siRNA.
(L) Percentages of cells with γH2AX foci after 8 hr JQ1 ± RAD51 siRNA (n = 4).
(M) Percentages of cells with 53BP1 foci after 8 hr JQ1 ± RAD51 siRNA (n = 4).
(N) Percentages of cells with γH2AX foci after JQ1 ± HEXIM1 siRNA (n = 3).
(O) Percentages of cells with 53BP1 foci after JQ1 ± HEXIM1 siRNA (n = 3).
(P) Model: BET inhibition disrupts recruitment of chromatin and replication factors; increased RNA synthesis and RAD51 activity (e.g., fork reversal) slow replication forks without DNA damage.
Data are represented as mean ± SEM. Scale bars, 10 μm. See also Figure S4.

  - Next-generation sequencing data analysis
  - Quantitative RT-PCR
  - Colony survival assay
  - Flow cytometry
- QUANTIFICATION AND STATISTICAL ANALYSIS
- DATA AND SOFTWARE AVAILABILITY

SUPPLEMENTAL INFORMATION

Supplemental Information includes four figures and one table and can be found with this article online at https://doi.org/10.1016/j.celrep.2018.10.079.

ACKNOWLEDGMENTS

We thank Drs. Catherine Rogers and Panagis Filippakopoulos for BRD4 expression vectors and Drs. Andy Turnell and Helen Stone for SMARCAL1 reagents. This work was supported by the Medical Research Council (MRC) (1552339), the German Research Foundation (PI 1300/1-1), the Wellcome Trust (202115/Z/16/Z), the Royal Society (RG170246), and a University of Birmingham fellowship to M.S.

AUTHOR CONTRIBUTIONS

A.B. designed and performed experiments, analyzed data, and contributed to writing the paper. A.L.P. performed RAD51 foci experiments. P.R. and M.S. analyzed NGS data. M.S. performed qRT-PCR. E.P. conceived the project, designed experiments, and wrote the paper.

DECLARATION OF INTERESTS

The authors declare no competing interests.

Received: July 6, 2018
Revised: September 27, 2018
Accepted: October 19, 2018
Published: November 20, 2018

REFERENCES

Andrieu, G., Belkina, A.C., and Denis, G.V. (2016). Clinical trials for BET inhibitors run ahead of the science. Drug Discov. Today. Technol. *19*, 45–50.

Bartholomeeusen, K., Xiang, Y., Fujinaga, K., and Peterlin, B.M. (2012). Bromodomain and extra-terminal (BET) bromodomain inhibition activate transcription via transient release of positive transcription elongation factor b (P-TEFb) from 7SK small nuclear ribonucleoprotein. J. Biol. Chem. *287*, 36609–36616.

Bhat, K.P., and Cortez, D. (2018). RPA and RAD51: fork reversal, fork protection, and genome stability. Nat. Struct. Mol. Biol. *25*, 446–453.

Byers, S.A., Price, J.P., Cooper, J.J., Li, Q., and Price, D.H. (2005). HEXIM2, a HEXIM1-related protein, regulates positive transcription elongation factor b through association with 7SK. J. Biol. Chem. *280*, 16360–16367.

Chaidos, A., Caputo, V., Gouvedenou, K., Liu, B., Marigo, I., Chaudhry, M.S., Rotolo, A., Tough, D.F., Smithers, N.N., Bassil, A.K., et al. (2014). Potent antimyeloma activity of the novel bromodomain inhibitors I-BET151 and I-BET762. Blood *123*, 697–705.

Da Costa, D., Agathanggelou, A., Perry, T., Weston, V., Petermann, E., Zlatanou, A., Oldreive, C., Wei, W., Stewart, G., Longman, J., et al. (2013). BET inhibition as a single or combined therapeutic approach in primary paediatric B-precursor acute lymphoblastic leukaemia. Blood Cancer J. *3*, e126.

Delmore, J.E., Issa, G.C., Lemieux, M.E., Rahl, P.B., Shi, J., Jacobs, H.M., Kastritis, E., Gilpatrick, T., Paranal, R.M., Qi, J., et al. (2011). BET bromodomain inhibition as a therapeutic strategy to target c-Myc. Cell *146*, 904–917.

Devaraj, S.G., Fiskus, W., Shah, B., Qi, J., Sun, B., Iyer, S.P., Sharma, S., Bradner, J.E., and Bhalla, K.N. (2016). HEXIM1 induction is mechanistically involved in mediating anti-AML activity of BET protein bromodomain antagonist. Leukemia *30*, 504–508.

Floyd, S.R., Pacold, M.E., Huang, Q., Clarke, S.M., Lam, F.C., Cannell, I.G., Bryson, B.D., Rameseder, J., Lee, M.J., Blake, E.J., et al. (2013). The bromodomain protein Brd4 insulates chromatin from DNA damage signalling. Nature *498*, 246–250.

Fujinaga, K., Luo, Z., Schaufele, F., and Peterlin, B.M. (2015). Visualization of positive transcription elongation factor b (P-TEFb) activation in living cells. J. Biol. Chem. *290*, 1829–1836.

Fujisawa, T., and Filippakopoulos, P. (2017). Functions of bromodomain-containing proteins and their roles in homeostasis and cancer. Nat. Rev. Mol. Cell Biol. *18*, 246–262.

Huang, M., Garcia, J.S., Thomas, D., Zhu, L., Nguyen, L.X., Chan, S.M., Majeti, R., Medeiros, B.C., and Mitchell, B.S. (2016). Autophagy mediates proteolysis of NPM1 and HEXIM1 and sensitivity to BET inhibition in AML cells. Oncotarget *7*, 74917–74930.

Ito, M., Yamamoto, S., Nimura, K., Hiraoka, K., Tamai, K., and Kaneda, Y. (2005). Rad51 siRNA delivered by HVJ envelope vector enhances the anti-cancer effect of cisplatin. J. Gene Med. *7*, 1044–1052.

Ketchart, W., Smith, K.M., Krupka, T., Wittmann, B.M., Hu, Y., Rayman, P.A., Doughman, Y.Q., Albert, J.M., Bai, X., Finke, J.H., et al. (2013). Inhibition of metastasis by HEXIM1 through effects on cell invasion and angiogenesis. Oncogene *32*, 3829–3839.

Kotsantis, P., Jones, R.M., Higgs, M.R., and Petermann, E. (2015). Cancer therapy and replication stress: forks on the road to perdition. Adv. Clin. Chem. *69*, 91–138.

Kotsantis, P., Silva, L.M., Irmscher, S., Jones, R.M., Folkes, L., Gromak, N., and Petermann, E. (2016). Increased global transcription activity as a mechanism of replication stress in cancer. Nat. Commun. *7*, 13087.

Lamoureux, F., Baud'huin, M., Rodriguez Calleja, L., Jacques, C., Berreur, M., Rédini, F., Lecanda, F., Bradner, J.E., Heymann, D., and Ory, B. (2014). Selective inhibition of BET bromodomain epigenetic signalling interferes with the bone-associated tumour vicious cycle. Nat. Commun. *5*, 3511.

Lew, Q.J., Chia, Y.L., Chu, K.L., Lam, Y.T., Gurumurthy, M., Xu, S., Lam, K.P., Cheong, N., and Chao, S.H. (2012). Identification of HEXIM1 as a positive regulator of p53. J. Biol. Chem. *287*, 36443–36454.

Lockwood, W.W., Zejnullahu, K., Bradner, J.E., and Varmus, H. (2012). Sensitivity of human lung adenocarcinoma cell lines to targeted inhibition of BET epigenetic signaling proteins. Proc. Natl. Acad. Sci. U S A *109*, 19408–19413.

Maruyama, T., Farina, A., Dey, A., Cheong, J., Bermudez, V.P., Tamura, T., Sciortino, S., Shuman, J., Hurwitz, J., and Ozato, K. (2002). A Mammalian bromodomain protein, brd4, interacts with replication factor C and inhibits progression to S phase. Mol. Cell. Biol. *22*, 6509–6520.

Muhar, M., Ebert, A., Neumann, T., Umkehrer, C., Jude, J., Wieshofer, C., Rescheneder, P., Lipp, J.J., Herzog, V.A., Reichholf, B., et al. (2018). SLAM-seq defines direct gene-regulatory functions of the BRD4-MYC axis. Science *360*, 800–805.

Pawar, A., Gollavilli, P.N., Wang, S., and Asangani, I.A. (2018). Resistance to BET inhibitor leads to alternative therapeutic vulnerabilities in castration-resistant prostate cancer. Cell Rep. *22*, 2236–2245.

Philpott, M., Rogers, C.M., Yapp, C., Wells, C., Lambert, J.P., Strain-Damerell, C., Burgess-Brown, N.A., Gingras, A.C., Knapp, S., and Müller, S. (2014). Assessing cellular efficacy of bromodomain inhibitors using fluorescence recovery after photobleaching. Epigenetics Chromatin *7*, 14.

Quaresma, A.J.C., Bugai, A., and Barboric, M. (2016). Cracking the control of RNA polymerase II elongation by 7SK snRNP and P-TEFb. Nucleic Acids Res. *44*, 7527–7539.

Sansam, C.G., Pietrzak, K., Majchrzycka, B., Kerlin, M.A., Chen, J., Rankin, S., and Sansam, C.L. (2018). A mechanism for epigenetic control of DNA replication. Genes Dev. *32*, 224–229.

Schröder, S., Cho, S., Zeng, L., Zhang, Q., Kaehlcke, K., Mak, L., Lau, J., Bisgrove, D., Schnölzer, M., Verdin, E., et al. (2012). Two-pronged binding with bromodomain-containing protein 4 liberates positive transcription elongation

factor b from inactive ribonucleoprotein complexes. J. Biol. Chem. *287*, 1090–1099.

Sørensen, C.S., Hansen, L.T., Dziegielewski, J., Syljuåsen, R.G., Lundin, C., Bartek, J., and Helleday, T. (2005). The cell-cycle checkpoint kinase Chk1 is required for mammalian homologous recombination repair. Nat. Cell Biol. *7*, 195–201.

Tan, J.L., Fogley, R.D., Flynn, R.A., Ablain, J., Yang, S., Saint-André, V., Fan, Z.P., Do, B.T., Laga, A.C., Fujinaga, K., et al. (2016). Stress from Nucleotide Depletion Activates the Transcriptional Regulator HEXIM1 to Suppress Melanoma. Mol. Cell *62*, 34–46.

Winter, G.E., Mayer, A., Buckley, D.L., Erb, M.A., Roderick, J.E., Vittori, S., Reyes, J.M., di Iulio, J., Souza, A., Ott, C.J., et al. (2017). BET bromodomain proteins function as master transcription elongation factors independent of CDK9 recruitment. Mol. Cell *67*, 5–18.e19.

Yang, L., Zhang, Y., Shan, W., Hu, Z., Yuan, J., Pi, J., Wang, Y., Fan, L., Tang, Z., Li, C., et al. (2017). Repression of BET activity sensitizes homologous recombination-proficient cancers to PARP inhibition. Sci. Transl. Med. *9*, 9.

Zellweger, R., Dalcher, D., Mutreja, K., Berti, M., Schmid, J.A., Herrador, R., Vindigni, A., and Lopes, M. (2015). Rad51-mediated replication fork reversal is a global response to genotoxic treatments in human cells. J. Cell Biol. *208*, 563–579.

Zhang, J., Dulak, A.M., Hattersley, M.M., Willis, B.S., Nikkilä, J., Wang, A., Lau, A., Reimer, C., Zinda, M., Fawell, S.E., et al. (2018). BRD4 facilitates replication stress-induced DNA damage response. Oncogene *37*, 3763–3777.

STAR★METHODS

KEY RESOURCES TABLE

REAGENT or RESOURCE	SOURCE	IDENTIFIER
Antibodies		
Rat monoclonal anti-BrdU [BU1/75]	Abcam	Cat# ab6326, RRID:AB_305426
Mouse monoclonal anti-BrdU [B44]	Becton Dickinson	Cat# 347580, RRID:AB_10015219
Mouse monoclonal anti-phospho-Histone H2AX (Ser139) [JBW301]	Millipore	Cat# 05-636, RRID:AB_309864
Rabbit polyclonal anti-53BP1	Bethyl	A300-272A, RRID:AB_185520
Mouse monoclonal anti-RNA Pol II pS2 [H5]	Abcam	at# ab24758, RRID:AB_2167352
Mouse monoclonal anti-RPA32 [RPA34-19]	Merck	Cat# NA18-100UG, RRID:AB_213121
Rabbit polyclonal anti-RAD51	Abcam	Cat# ab63801, RRID:AB_1142428
Rabbit monoclonal anti-BRD2 [EPR7642]	Abcam	Cat# ab139690, RRID:AB_2737409
Rabbit polyclonal anti-BRD3	Abcam	Cat# ab83478, RRID:AB_1860012
Rabbit monoclonal anti-BRD4 [EPR5150(2)]	Abcam	Cat# ab128874, RRID:AB_11145462
Rabbit polyclonal anti-HEXIM1	Abcam	Cat# ab25388, RRID:AB_2233058
Rabbit polyclonal anti-pS4/S8 RPA32	Bethyl	Cat# A300-245A, RRID:AB_210547
Rabbit polyclonal anti-pS317 CHK1	Cell Signaling	Cat# 2344, RRID:AB_331488
Mouse monoclonal anti-PARP1 [F-2]	Santa Cruz	Cat# sc-8007, RRID:AB_628105
Rabbit polyclonal anti-ZRANB3	Proteintech	Cat# 23111-1-AP RRID:AB_2744527
Mouse monoclonal anti-SMARCAL1 [A-2]	Santa Cruz	Cat# sc-376377 RRID:AB_10987841
Mouse monoclonal anti-αTUBULIN [B512]	Sigma	Cat# T6074, RRID:AB_477582
Rabbit polyclonal anti-βACTIN	Cell Signaling	Cat# 4967, RRID:AB_330288
Chemicals, Peptides, and Recombinant Proteins		
(+)-JQ1	Tocris	4499/10
I-BET151	Tocris	4650/10
AZ20	Tocris	5198/10
Olaparib	Selleckchem	S1060
Triptolide	Tocris	3253/1
DRB	Sigma	D1916
alpha-Amanitin	Sigma	A2263
Hydroxyurea	Sigma	H8627
Critical Commercial Assays		
Click-iT RNA Alexa Fluor 594 Imaging Kit	Thermo Fisher	C10330
RNeasy Mini Kit	QIAGEN	74104
miRNeasy Mini Kit	QIAGEN	217004
SuperScript III Reverse Transcriptase	Thermo Fisher	18080093
SensiFAST SYBR Lo-ROX Kit	Bioline	BIO-94005
Deposited Data		
Gene Expression Omnibus Series GSE111463	Muhar et al., 2018	https://www.ncbi.nlm.nih.gov/geo/query/acc.cgi?acc=GSE111463
Experimental Models: Cell Lines		
U2OS	ATCC	Cat# HTB-96, RRID:CVCL_0042
BJ-hTert	ATCC	Cat# CRL-4001, RRID:CVCL_6573
NALM-6	DSMZ	Cat# ACC-128, RRID:CVCL_0092
C2	DSMZ	Cat# ACC-773, RRID:CVCL_0D73
MEC1	DSMZ	Cat# ACC-497, RRID:CVCL_1870

(*Continued on next page*)

Continued

REAGENT or RESOURCE	SOURCE	IDENTIFIER
Oligonucleotides		
BRD2 ON-TARGETplus SMARTpool - Human	Dharmacon	L-004935-00
BRD3 ON-TARGETplus SMARTpool - Human	Dharmacon	L-004936-00
BRD4 ON-TARGETplus SMARTpool - Human	Dharmacon	L-004937-00
HEXIM1 ON-TARGETplus SMARTpool - Human	Dharmacon	L-012225-01
RAD51 siRNA, targeting sequence 5′-GAGCUUGACAAACUACUUC-3′	Ito et al., 2005	n/a
ZRANB3 individual siGENOME siRNA	Dharmacon	D-010025-03
SMARCAL1 siRNA	Dharmacon	L-013058-00
Allstars negative control siRNA	QIAGEN	SI03650318
See Table S1 for qRT-PCR primers	n/a	n/a
Recombinant DNA		
pcDNA6.2/N-EmGFP-BRD4(long)-DEST	Philpott et al., 2014	n/a
pCDNA5-3HA-BRD4(short)-DEST	Gift from Panagis Fillipakopoulos	n/a
pcDNA3.1/V5/His-TOPO-RAD51	Sørensen et al., 2005	n/a
pcDNA3.1(+)	ThermoFisher	V79020
pEGFP-C2	Clontech	6083-1
Software and Algorithms		
ImageJ	n/a	https://imagej.nih.gov/ij/
ENCODE tools	n/a	https://www.encodeproject.org/software/wigtobigwig/
deepTools	n/a	http://deeptools.ie-freiburg.mpg.de/
Integrative Genomics Viewer	n/a	https://software.broadinstitute.org/software/igv/

CONTACT FOR REAGENT AND RESOURCE SHARING

Further information and requests for resources and reagents should be directed to and will be fulfilled by the Lead Contact, Eva Petermann (e.petermann@bham.ac.uk).

EXPERIMENTAL MODEL AND SUBJECT DETAILS

Human U2OS, NALM-6, C2, MEC1 and BJ-hTert cells (ATCC) were authenticated using 8-locus STR profiling (LGC Standards). Cells were confirmed Mycoplasma free and grown in DMEM (high glucose) or RPMI 1640 in a humidified atmosphere containing 5% CO2.

METHOD DETAILS

Drug treatments

JQ1 (1 μM), I-BET151 (1 μM), AZ20 (2.4 μM), and triptolide (1 μM) were from Tocris Bioscience. Olaparib (5 μM) was from Selleckchem. 5,6-dichloro-1-β-D-ribofuranosyl-1H-benzimidazole (DRB, 100 μM), α-amanitin (10 μg/ml), and hydroxyurea (2 mM) were from Sigma.

DNA fiber analysis

Cells were labeled with 25 μM CldU and 250 μM IdU for 20 min each and DNA fiber spreads prepared. HCl-treated fiber spreads were incubated with rat anti-BrdU (BU1/75, Abcam ab6326, 1:250) and mouse anti-BrdU (B44, Becton Dickinson 347580, 1:500) for 1 h, fixed with 4% PFA and incubated with anti-rat AlexaFluor 555 and anti-mouse AlexaFluor 488 (Thermo Fisher) for 1.5 h.

EU incorporation assay

5-ethynyl uridine (EU) incorporation assays were performed using the Click-iT RNA Alexa Fluor 594 Imaging Kit (Thermo Fisher). Cells were incubated with 1 mM EU for 1 h, followed by fixation and Click reaction according to manufacturer's instructions. DNA was counterstained with DAPI. Imaging was performed immediately after Click reaction and nuclear masks were generated in ImageJ

to quantify mean fluorescence intensities per nucleus. Results were normalized to control/DMSO to account for variation in staining intensity.

siRNA and DNA transfection

siRNAs against BRD2, BRD3, BRD4, HEXIM1, SMARCAL1, ZRANB3, (L-004935-00, L-004936-00, L-004937-00, L-012225-01, L-013058-00, D-010025-03), and RAD51 (Ito et al., 2005), were from Dharmacon, and "Allstars negative control siRNA" from QIAGEN. Cells were transfected with 50 nM siRNA using Dharmafect 1 reagent (Dharmacon). For BRD4 and RAD51 overexpression cells were transfected using TransIT-2020 (Mirus Bio) with 2.5 μg pcDNA6.2/N-EmGFP-BRD4(long)-DEST (Philpott et al., 2014), pCDNA5-3HA-BRD4(short)-DEST (kind gift from Dr Panagis Filippakopoulos), pcDNA3.1/V5/His-TOPO-RAD51 (Sørensen et al., 2005), or control plasmids pcDNA3.1(+) (Thermo Fisher) or pEGFP-C2 (Clontech).

Immunofluorescence

Cells were fixed with 4% PFA for 10 min, permeabilised with 0.25% Triton X-100 for 5 min, and blocked with 2% BSA. Primary antibodies were mouse anti-phospho-Histone H2AX (Ser139) (JBW301, Millipore 05-636, 1:1000), rabbit anti-53BP1 (Bethyl A300-272A, 1:30000), mouse anti-RNA Pol II pS2 (Abcam ab24758, 1:500,), mouse anti-RPA32 (Merck NA18, 1:500) and rabbit anti-RAD51 (Abcam ab63801, 1:500). Secondary antibodies were anti-mouse IgG AlexaFluor 488 and anti-rabbit IgG AlexaFluor 555 (Thermo Fisher). DNA was counterstained with DAPI and images were acquired on a Nikon E600 microscope with a Nikon Plan Apo 60x (1.3 NA) oil lens, a Hamamatsu digital camera (C4742-95) and the Volocity acquisition software (Perkin Elmer). Images were analyzed using ImageJ. Cells with more than 5 RAD51 foci and 8 γH2AX or 53BP1 foci were scored as positive. Scale bars are 10 μm.

Western blotting

Cell extracts were prepared in UTB buffer (50 mM Tris-HCl pH 7.5, 150 mM β-mercaptoethanol, 8 M urea) and sonicated to release DNA-bound proteins. Primary antibodies used were rabbit anti-BRD2 (Abcam ab139690, 1:1000), rabbit anti-BRD3 (Abcam ab83478, 1:500), rabbit anti-BRD4 (Abcam ab128874, 1:1000), rabbit anti-HEXIM1 (Abcam ab25388,1:5000), rabbit anti-pS4/S8 RPA32 (Bethyl A300-245A, 1:1000), rabbit anti-pS317 CHK1 (Cell Signaling 2344, 1:1000), rabbit anti-RAD51 (Abcam, ab63801, 1:1000) mouse anti-PARP1 (Santa Cruz sc8007, 1:1000), rabbit anti-ZRANB3 (Proteintech 23111-1-AP, 1:500), mouse anti-SMARCAL1 (Santa Cruz sc-376377, 1:15,000), mouse anti-αTUBULIN (B512, Sigma T6074, 1:10000), rabbit anti-βACTIN (Cell Signaling 4967, 1:5000).

RNA isolation

Cells were harvested and counted, and RNA was isolated using the QIAGEN RNeasy Mini Kit according to manufacturer's instructions. RNA concentration was determined using an Implen NanoPhotometer Pearl and normalized to cell numbers.

Next-generation sequencing data analysis

The available bigwig RNA Pol II ChIP-Seq files were downloaded and analyzed using ENCODE tools (https://www.encodeproject.org/software/wigtobigwig/) and deepTools (http://deeptools.ie-freiburg.mpg.de/) to obtain files for the quantification analysis. To identify genes with more RNA Pol II in the dataset (IAA versus DMSO), average scores in every genomic region were calculated for each bigWig file using deepTools. The Integrative Genomics Viewer (IGV) snapshots were generated loading the respective bigwig files on IGV, while the difference between the coverages was generated using igvtools/combine tracks.

Quantitative RT-PCR

Total RNA was harvested using the miRNeasy Mini Kit (QIAGEN) followed by DNase I treatment (Roche). 1 μg of total RNA was reverse-transcribed using SuperScript Reverse Transcriptase III (ThermoFisher) with random primers (Promega), following manufacturer's instructions. The qPCR primers for amplification are listed in Table S1. For quantitative RT-PCR, 2 μl of cDNA were analyzed using a CFX Connect real-time PCR machine (BioRad) with SensiFAST SYBR Lo-ROX Kit (Bioline). Cycling parameters were 95°C for 3 min, followed by 40 cycles of 95°C for 10 s, 60°C for 30 s. Result were normalized to RPLP0, which we have found to be a very stable transcript. ΔcT was calculated as difference in the cycle threshold of the transcript of interest and RPLP0, plotted as fold change compared to the CTR untreated sample.

Colony survival assay

Defined numbers of cells were plated in duplicate before treatment with JQ1 (1 – 30 μM) for 24 h. Colonies of > 50 cells were allowed to form in fresh medium and fixed in 50% ethanol, 2% methylene blue.

Flow cytometry

Cells were fixed with cold 70% ethanol before staining with 10 μg/ml propidium iodide. Cell cycle profiles were gathered using the BD LSR Fortessa X20 and analyzed with BD FacsDiva software.

QUANTIFICATION AND STATISTICAL ANALYSIS

Values represent the means ± 1x SEM of independent biological repeats. For DNA fiber analysis, at least 100 fibers from 10 different areas were measured and the median of the distribution was calculated for each independent biological repeat. For foci and EU analysis, at least 10 different areas were quantified for each independent biological repeat. The number of independent biological repeats (n) is indicated in the figure legends. The statistical test used throughout was the one-tailed Student's t test except for colony assays, where 2-way ANOVA with Tukey's was calculated using GraphPad Prism 6, as indicated in the figure legends. Asterisks compare to control, unless indicated otherwise in the figure panels, and signify $*p < 0.05$, $**p < 0.01$, $***p < 0.001$, $****p < 0.0001$.

DATA AND SOFTWARE AVAILABILITY

This study analyzed published next generation sequencing data (Muhar et al., 2018). The accession number for these data is GEO: GSE111463.

Cell Reports, Volume *25*

Supplemental Information

BET Inhibition Induces HEXIM1- and RAD51-Dependent Conflicts between Transcription and Replication

Akhil Bowry, Ann Liza Piberger, Patricia Rojas, Marco Saponaro, and Eva Petermann

Supplemental Figures

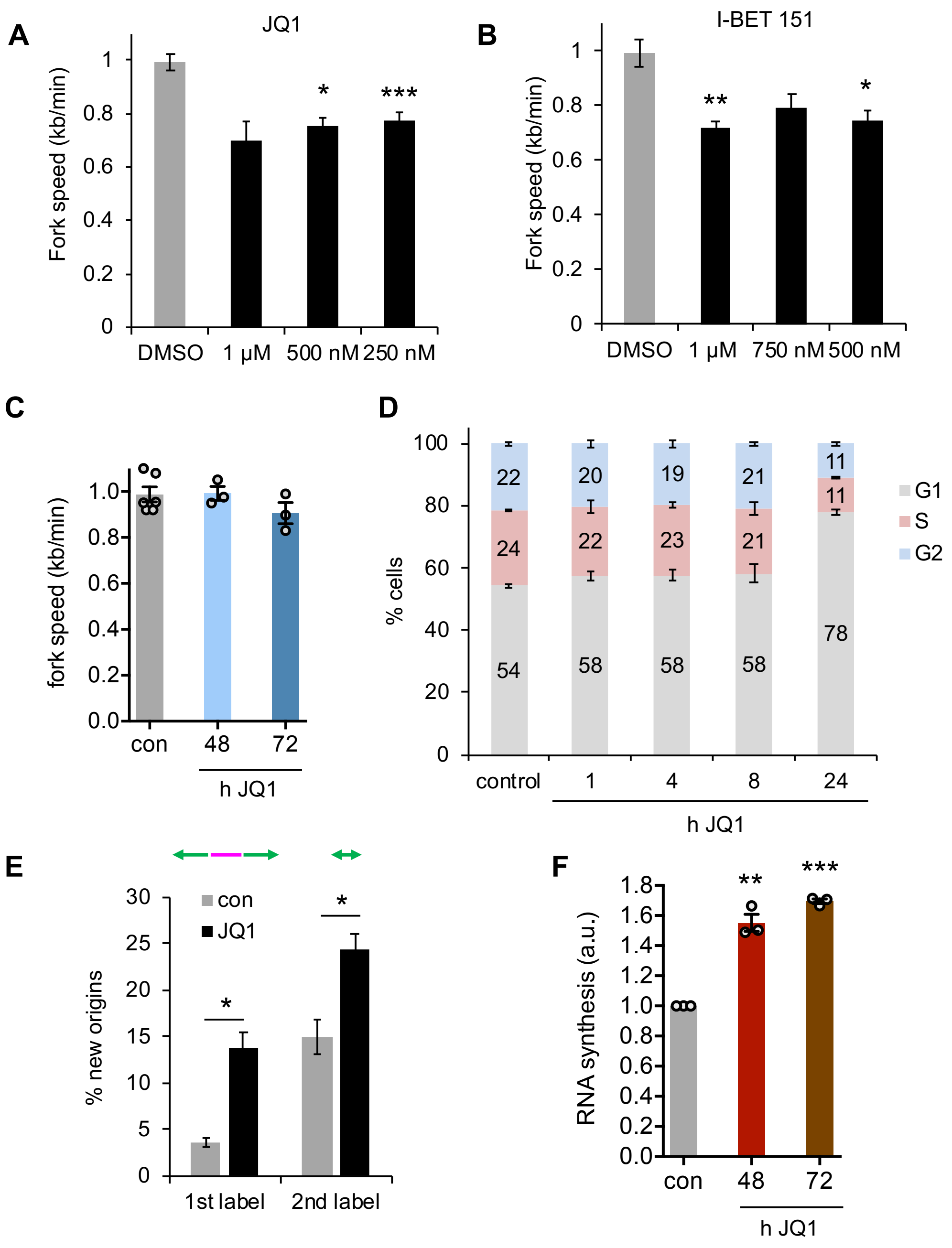

Figure S1. Related to Figure 1. BET inhibition induces replication fork slowing. A) Replication fork speeds in U2OS cells after treatment with 0.25-1 µM JQ1 for 1 h. n=3. B) Replication fork speeds in U2OS cells after treatment with 0.5-1 µM I-BET151 for 1 h. n=4. C) Replication fork speeds in U2OS cells treated with 1 µM JQ1 for 48 and 72 h. n=3. D) Cell cycle distribution measured by flow cytometry of U2OS cells after 1-24 h treatment with 1 µM JQ1. n=3. E) New origin firing in U2OS cells after treatment with 1 µM JQ1 for 1 h. n=3. F) Nuclear EU intensity in U2OS cells treated with 1 µM JQ1 for 48 and 72 h. n=3. Data are represented as mean ± SEM.

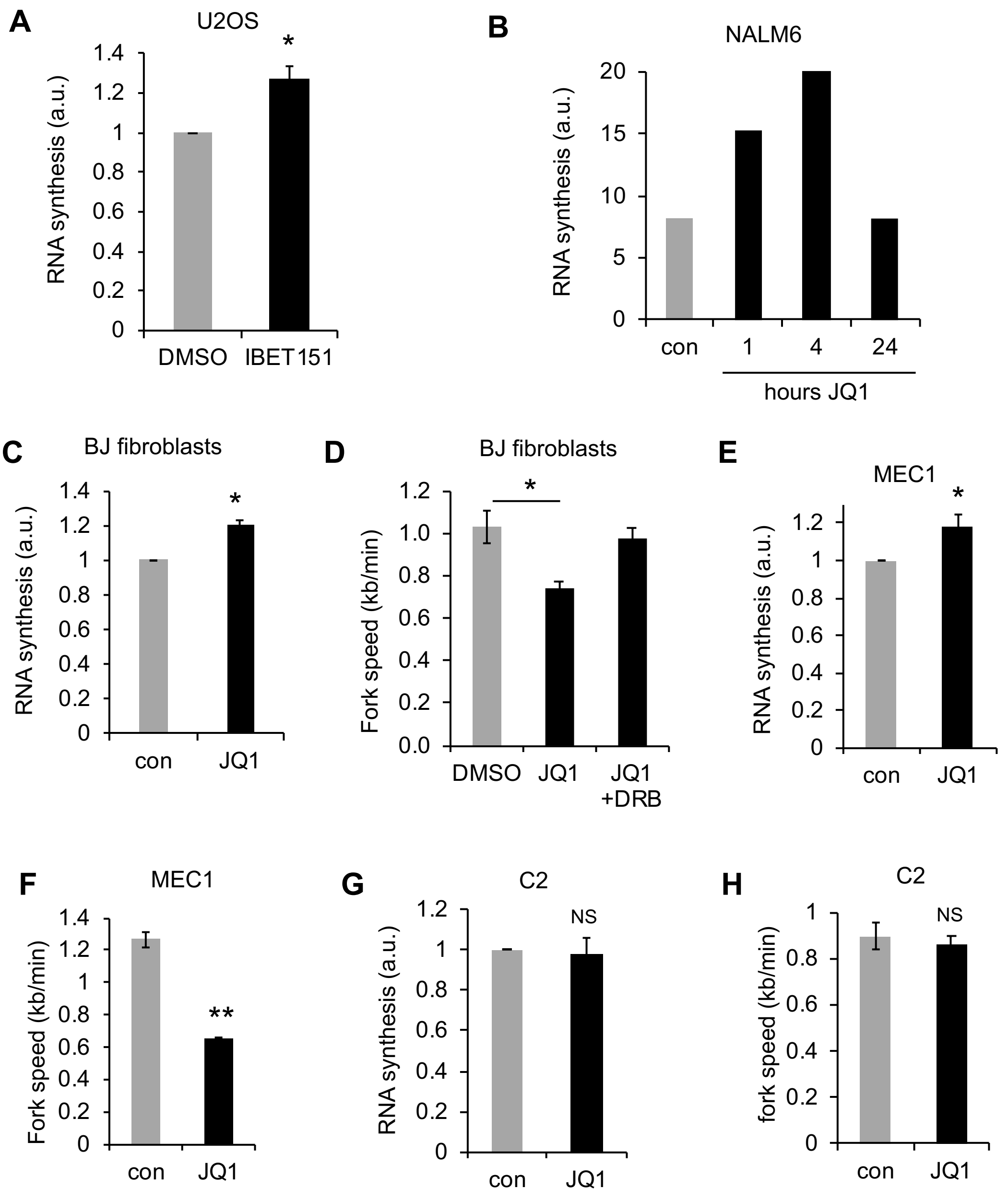

Figure S2. Related to Figure 1. Replication-transcription conflicts induced by I-BET151 and in a number of cell lines. A) Quantification of nuclear EU intensity in U2OS cells after 1 µM I-BET151 (1h). n=4. B) Nuclear EU intensity in NALM6 cells after 1-24h 1 µM JQ1. n=1. C) Nuclear EU intensity in BJ-hTert fibroblasts +/- 1 µM JQ1 (1h). n=3. D) Median replication fork speeds in BJ-hTert fibroblasts +/- 1 µM JQ1 (1h), and treated with JQ1 and DRB for 1h. n=3. E) Nuclear EU intensity in MEC1 cells +/-1 µM JQ1 (1h). n=3. F) Median replication fork speeds in MEC1 cells +/- 1 µM JQ1 (1h). n=5. G) Nuclear EU intensity in C2 cells +/-1 µM JQ1 (1h). n=3. H) Median replication fork speeds in C2 cells +/- 1 µM JQ1 (1h). n=3. Data are represented as mean ± SEM.

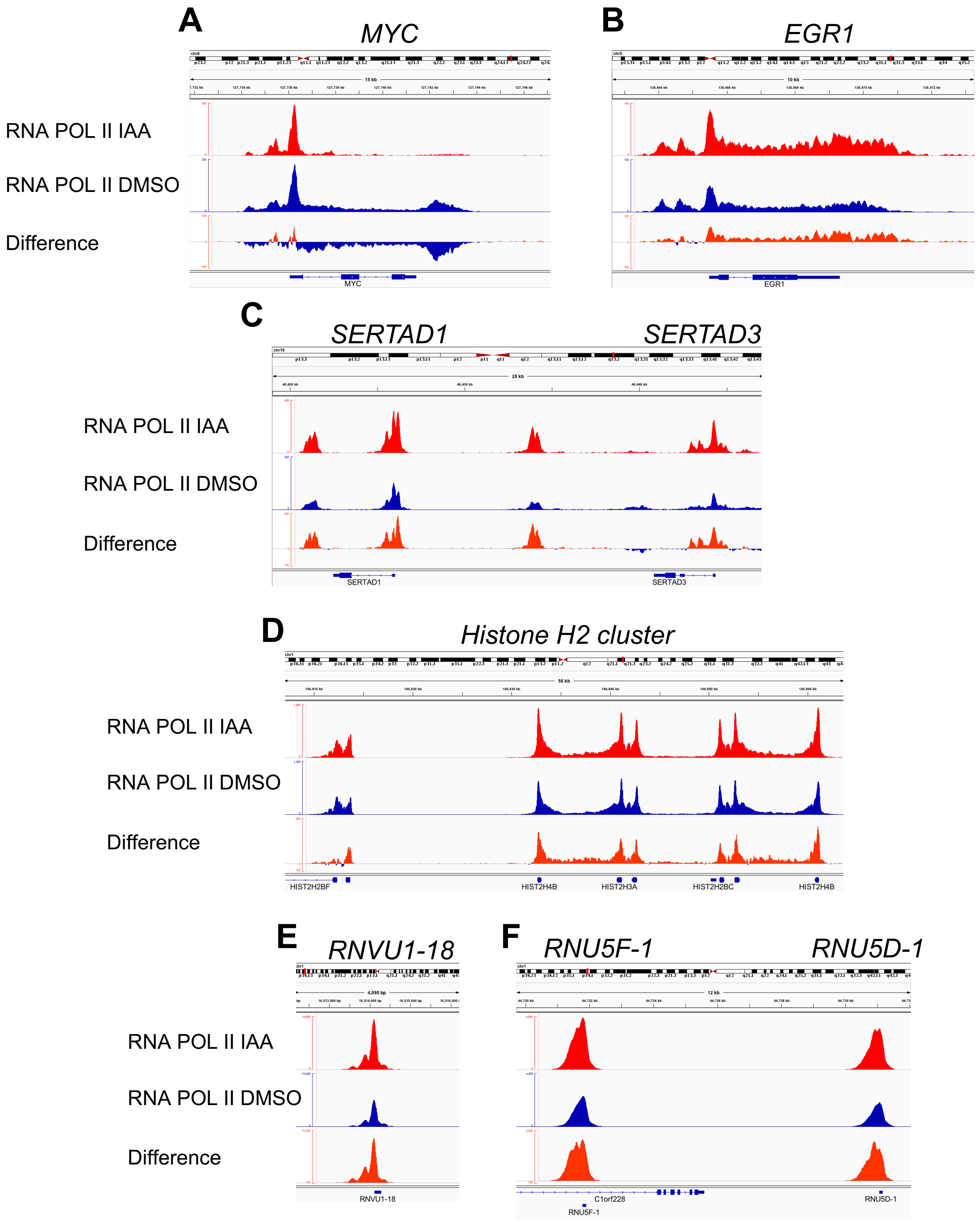

Figure S3. Related to Figure 1. BRD4 degradation increases RNA Pol II occupancy in histone and non-coding RNA genes. Analysis of published RNA Pol II ChIP-seq data sets (Muhar et al., 2018; GEO Series GSE111463). RNA Pol II ChIP was performed 1h after BRD4 degradation using auxin inducible degron (AID) approach in K562 leukaemia cells. The mRNA expression of *MYC*, *EGR1* and *SERTAD1* was analysed in parallel (Muhar et al., 2018). A) *MYC* (mRNA decreased 1h after BRD4 degradation), B) *EGR1* (mRNA increased 1h after BRD4 degradation), C) *SERTAD1* (mRNA increased 1h after BRD4 degradation), D) histone gene cluster (non-polyadenylated transcripts), also with large increase in RNA Pol II levels outside genes boundaries, E) non-coding RNA *RNVU1-18* (non-polyadenylated transcript), F) non-coding RNAs *RNU5F-1* and *RNU5D-1* (non-polyadenylated transcripts). Data are represented as mean ± SEM.

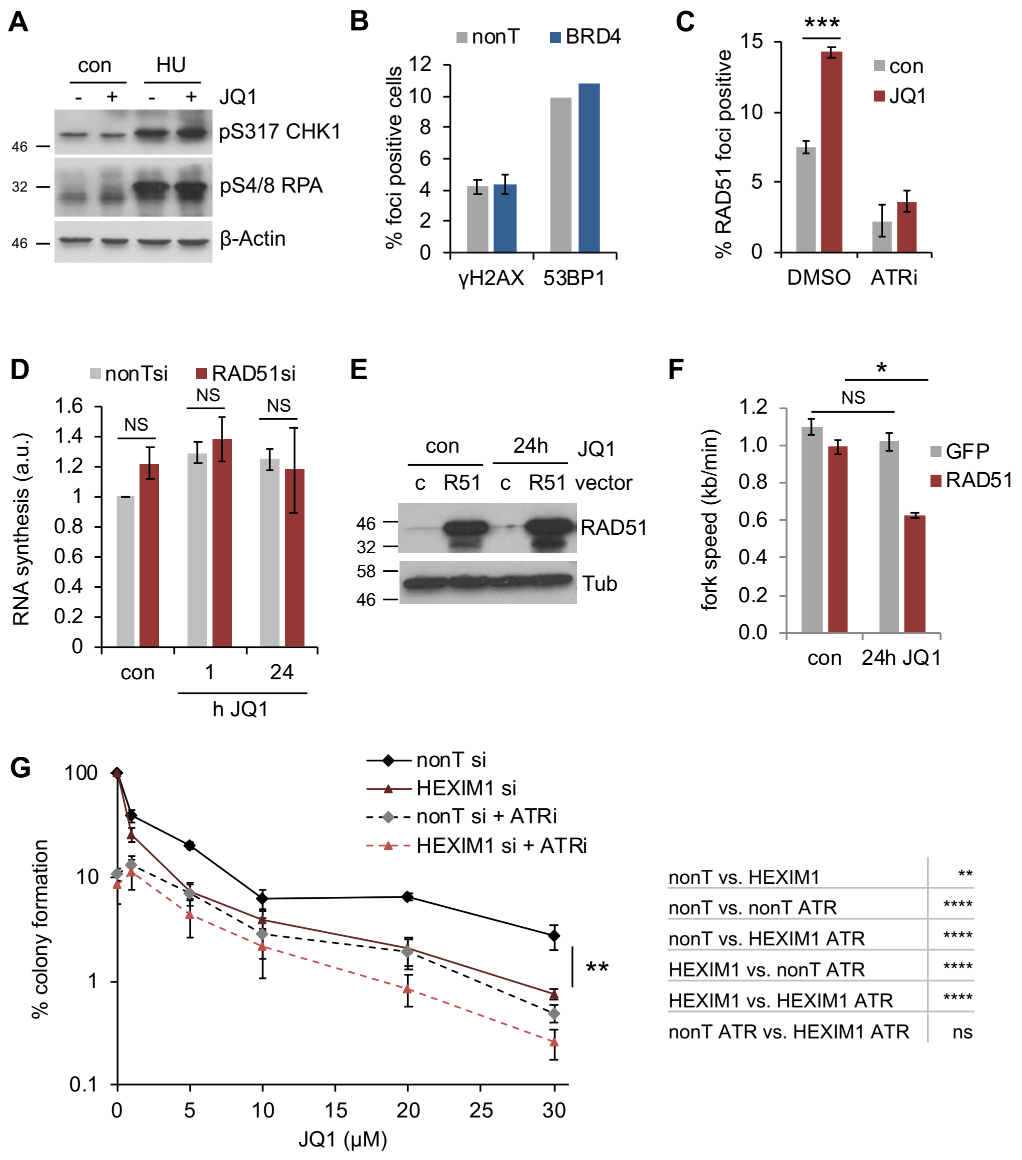

Figure S4. Related to Figure 4. DNA damage response to BET inhibition. A) Levels of phospho-S4/8 RPA32, phospho-S317 CHK1 and β-Actin after treatment with JQ1 and HU as indicated. B) Percentages of cells containing more than 8 γH2AX or 53BP1 foci after BRD4 depletion. n = 2 (γH2AX), n = 1 (53BP1). C) Percentages of cells containing more than 8 RAD51 foci after JQ1 treatment +/- ATR inhibitor AZ20. n=3 (DMSO), n=2 (ATRi). D) Quantification of nuclear EU intensity +/- RAD51 siRNA and JQ1 treatment. n=3. E) Levels of RAD51 and loading control after transient overexpression of RAD51 or eGFP (control) for 24 h +/- JQ1. F) Replication fork speeds after transient overexpression of RAD51 or eGFP (control) for 24 h +/- JQ1. n=3. G) Colony survival of U2OS cells treated with indicated concentrations of JQ1 for 24h, +/- HEXIM1 siRNA and AZ20. n=3. The statistical test used was 2-way ANOVA with Tukey's. Data are represented as mean ± SEM.

Supplementary Table S1. Related to STAR Methods. Sequences of PCR primers

Name	Sequence (5' → 3')
HIST2H3D For	CGAGATCGCGCAGGACTTTA
HIST2H3D Rev	TGTCCTTGGGCATGATGGTC
HIST2H4B For	GAAGCTGTCTATCGGGCTCC
HIST2H4B Rev	ACTGCGTCCCGAATCACATT
HIST2H2BE For	GCCACCCACCTAATCACTAGAAA
HIST2H2BE Rev	CAATGACGCACTGGGGACC
HIST4H4 For	GGGTCGCACCCTTTATGGTT
HIST4H4 Rev	GTATCCGGAAGGCGACATCA
EGR1 For	TGCAGATCTCTGACCCGTTC
EGR1 Rev	CAGGAAAAGACTCTGCGGTCA
SERTAD1 For	GAGGACAGCCAACAAGCGAT
SERTAD1 Rev	TGCTCAGCATCTTGCTCACTA
MYC For	CAGCGACTCTGAGGAGGAAC
MYC Rev	GCTGCGTAGTTGTGCTGATG
RNU5D1 For	GCTCTGGTTTCTCTTCAAAT
RNU5D1 Rev	AACCCCACCAACATAGGG
RNVU1-18 For	GGTTTTCCCAGGGCGAGG
RNVU1-18 Rev	CCACTACCACAAATTATGCAGTC
RPLP0 F2 (control)	CAGATTGGCTACCCAACTGTT
RPLP0 R2 (control)	GGAAGGTGTAATCCGTCTCCAC
